# Eosin Y-catalyzed visible-light-mediated aerobic oxidative cyclization of *N*,*N*-dimethylanilines with maleimides

**DOI:** 10.3762/bjoc.11.48

**Published:** 2015-04-01

**Authors:** Zhongwei Liang, Song Xu, Wenyan Tian, Ronghua Zhang

**Affiliations:** 1 Department of Chemistry, Tongji University, Siping Road 1239, Shanghai 200092, China; 2 Key Laboratory of Yangtze River Water Environment, Ministry of Education, Siping Road 1239, Shanghai 200092, China; 3 College of Biological, Chemical Sciences and Engineering, Jiaxing University, Jiahang Road 118, Zhejiang 314001, China

**Keywords:** aerobic oxidative cyclization, C–H functionalization, Eosin Y, photoredox catalysis, visible light

## Abstract

A novel and simple strategy for the efficient synthesis of the corresponding tetrahydroquinolines from *N*,*N*-dimethylanilines and maleimides using visible light in an air atmosphere in the presence of Eosin Y as a photocatalyst has been developed. The metal-free protocol involves aerobic oxidative cyclization via sp^3^ C–H bond functionalization process to afford good yields in a one-pot procedure under mild conditions.

## Introduction

Over the past several years, visible light photoredox catalysis has become a powerful and promising tool and has been productively used to drive chemical transformations in the field of organic synthesis [1–6]. The approach takes full advantage of visible light, which is clean, abundant, and renewable. The pioneering work in this research area, reported by the groups of MacMillan [7–9], Yoon [10–11], Stephenson [12–13] and others [14–18], has demonstrated that ruthenium and iridium complexes as visible light photoredox catalysts are capable of catalyzing a broad range of useful reactions. A variety of new methods have been developed to accomplish known and new chemical transformations by means of these transition metal-based photocatalysts so far.

However, the ruthenium and iridium catalysts usually are high-cost, potentially toxic and not sustainable. Similar to the redox properties of these organometallic complexes, some metal-free organic dyes such as Eosin Y, Rose Bengal, Fluorescein, and Methylene Blue, have shown superiority of their applications as photocatalysts, which are easy to handle, environmentally friendly, inexpensive, and have great potential for applications in visible-light-mediated photoredox reactions [19–27].

**Scheme 1:**
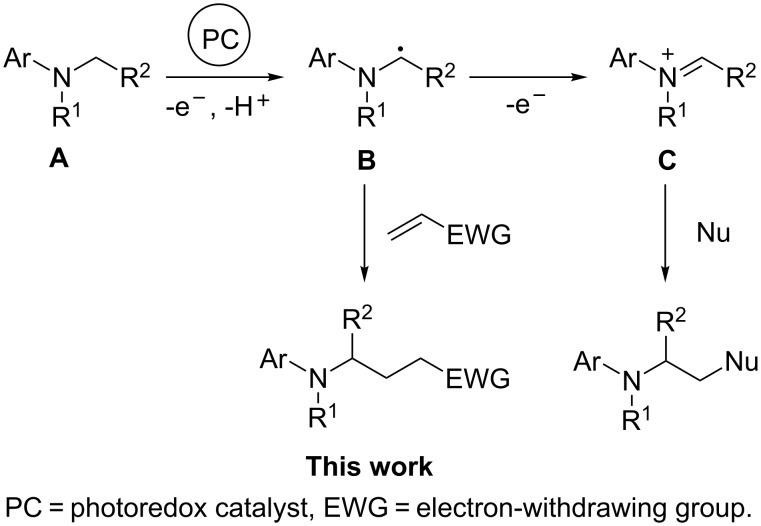
Visible-light-induced sp^3^ C–H bond functionalization of tertiary amines.

More recently, visible-light-induced sp^3^ C–H bond functionalization adjacent to nitrogen atoms has been extensively studied and has become a fundamental organic transformation [28–38]. Tertiary amine **A** generally generates a nucleophilic α-aminoalkyl radical **B** or an electrophilic iminium ion **C** via visible-light photoredox catalysis. Unfortunately the research of the α-aminoalkyl radical is limited in photochemical synthesis because it tends to form the iminium ion by one electron oxidation (Scheme 1) [39–41].

In the context of this research background, we investigated the α-aminoalkyl radical route to achieve the aerobic oxidative cyclization of *N*,*N*-dimethylanilines with maleimides to form the corresponding tetrahydroquinoline derivatives under organic dye Eosin Y catalysis. Swan and Roy reported the reaction using benzoyl peroxide as catalyst at low temperature as early as 1968 [42]. In 2011, Miura and co-workers achieved this transformation using a copper catalyst and air as the terminal oxidant [43]. Bian and co-workers presented the same reaction using [Ru(bpy)_3_]^3+^ as photoredox catalyst under irradiation with visible light next year [44]. Herein, we show an environmentally friendly aerobic oxidative cyclization methodology that avoids the use of metal catalysts and makes full use of air as oxidant.

## Results and Discussion

Our investigations for the envisaged protocol commenced with the reaction of *N*,*N*-dimethylaniline (**1a**) (0.5 mmol) with *N*-phenylmaleimide (**2a**) (0.25 mmol) in MeCN (3 mL) in the presence of 3 mol % Eosin Y under an air atmosphere (with no air bubbling). The reaction mixture was irradiated with visible light (two 9 W blue LEDs) at room temperature. Gratifyingly, the desired product tetrahydroquinoline **3a** was obtained in 82% yield after 18 h (Table 1, entry 1). We screened a number of metal-free organic dyes for photocatalysts. Of the dyes screened, Eosin Y showed the highest efficiency (Table 1, entry 1), Rose Bengal gave a slightly lower yield (Table 1, entry 2), whereas Methylene Blue and Fluorescein gave poor yields (Table 1, entries 3 and 4). Under an O_2_ atmosphere, the yield of tetrahydroquinoline product **3a** was decreased to 77% (Table 1, entry 5). When the molar proportion of **1a** and **2a** was adjusted to 1:1 and 1:2, the yield of **3a** decreased (Table 1, entries 6 and 7). Then, a series of control experiments were carried out, which indicated that Eosin Y, visible light and air are all essential for the reaction (Table 1, entries 8–10).

**Table 1:** Screening and control experiments^a^.

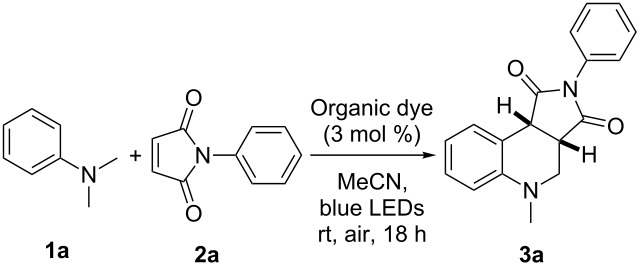			

Entry	Organic dye	Oxidant	Yield (%)^b^

1	Eosin Y	air	82
2	Rose Bengal	air	67
3	Methylene Blue	air	trace
4	Fluorescein	air	trace
5	Eosin Y	O_2_^c^	77
6	Eosin Y	air	69^d^
7	Eosin Y	air	73^e^
8	none	air	n.r.
9	Eosin Y	air	n.r.^f^
10	Eosin Y	none	trace^g^

^a^Reaction conditions: **1a** (0.5 mmol), **2a** (0.25 mmol), organic dye (3 mol %), MeCN (3 mL), two 9 W Blue LEDs irradiation under an air atmosphere at rt. ^b^Isolated yield of the product **3a**; n.r. = no reaction. ^c^Under O_2_ (1 atm, balloon). ^d^0.25 mmol of **1a** and 0.25 mmol of **2a** were used. ^e^0.25 mmol of **1a** and 0.5 mmol of **2a** were used. ^f^The reaction was carried out in the dark. ^g^Under N_2_.

Next, we optimized the reaction conditions with respect to solvent and catalyst dosage. MeCN was found to be the best solvent (Table 2, entry 1) among DMF, DCE, DCM, DMSO, acetone, dioxane, and MeNO_2_. When the amount of Eosin Y was decreased from 3 mol % to 2 mol % or increased from 3 mol % to 4 mol %, the yield of the tetrahydroquinoline product **3a** was slightly reduced (Table 2, entries 9 and 10). Thus, the optimum catalyst dosage of Eosin Y was found to be 3 mol %.

**Table 2:** Optimization of reaction conditions^a^.

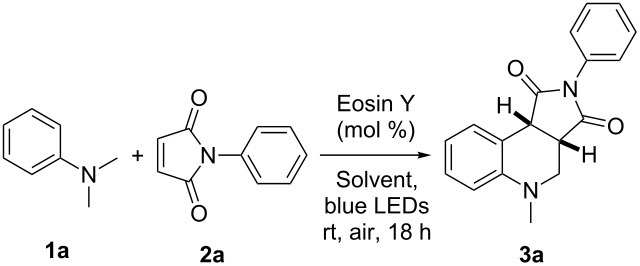			

Entry	Eosin Y (mol %)	Solvent	Yield (%)^b^

1	3	MeCN	82
2	3	DMF	47
3	3	DCE	71
4	3	DCM	37
5	3	DMSO	trace
6	3	acetone	64
7	3	dioxane	51
8	3	MeNO_2_	58
9	2	MeCN	80
10	4	MeCN	77

^a^Reaction conditions: **1a** (0.5 mmol), **2a** (0.25 mmol), solvent (3 mL), two 9 W blue LEDs irradiation under an air atmosphere at rt. ^b^Isolated yield of the product **3a**.

**Scheme 2:**
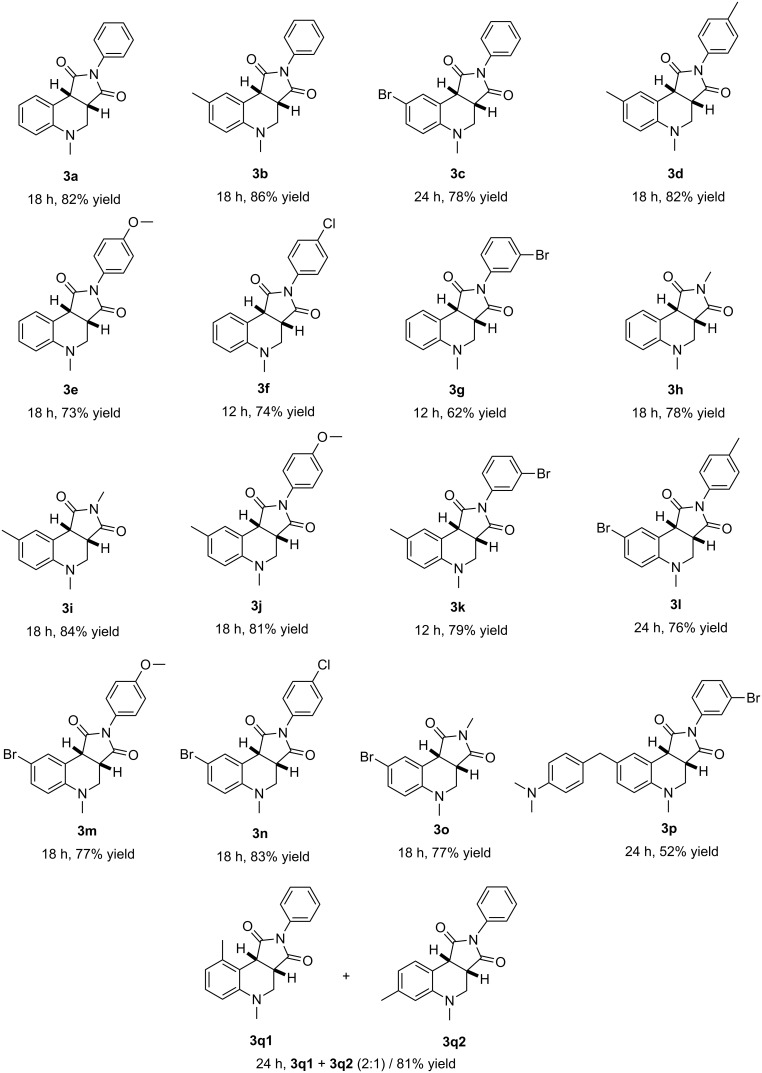
Substrate scope for aerobic oxidative cyclization of *N*,*N*-dimethylanilines with maleimides.

With the optimized conditions in hand, the substrate scope of this reaction was examined (Scheme 2). The reaction is mild and tolerates many functional groups. *N*,*N*-dimethylaniline and substituted *N*,*N*-dimethylanilines incorporating methyl and bromo on the phenyl ring reacted with **2** to afford the corresponding tetrahydroquinolines **3** in good yields. *N*-arylmaleimides with electron-donating groups such as methyl, methoxy and electron-withdrawing groups such as chloro, bromo, and *N*-methylmaleimide all underwent the aerobic oxidative cyclization to give the corresponding products in good yields. When using 4,4'-methylenebis(*N*,*N*-dimethylaniline) as the substrate, the reaction occurred only on one side and the yield of the product **3p** is 52%. The reaction of *N*,*N*,3-trimethylaniline and *N*-phenylmaleimide resulted in the formation of a mixture of regioisomers **3q1** and **3q2** with 81% combined yield. The major product was the sterically more hindered **3q1** [43–45].

**Scheme 3:**
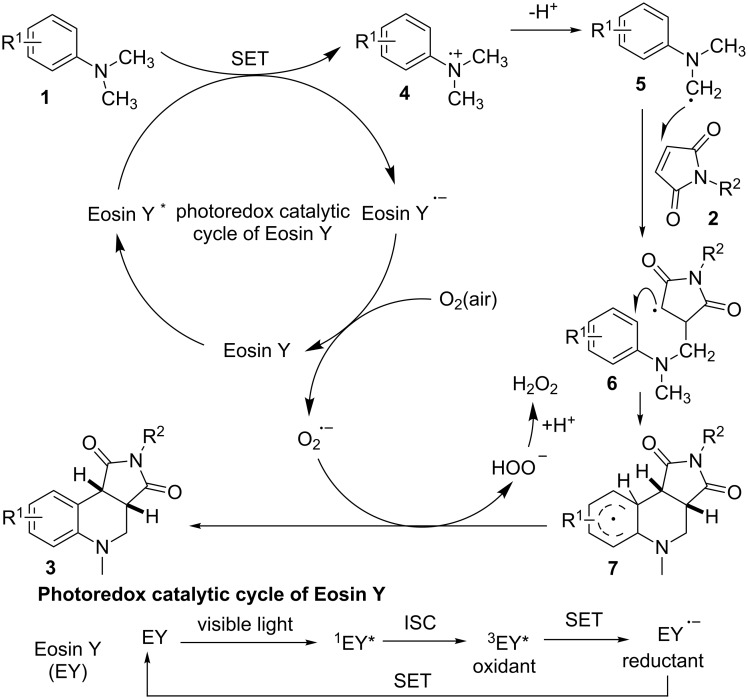
A proposed reaction mechanism.

On the basis of our observations and literature reported [19,36,38,43–44], a proposed mechanism for the formation of the corresponding tetrahydroquinolines **3** form *N*,*N*-dimethylanilines **1** and maleimides **2** is depicted in Scheme 3. On absorption of visible light, the ground state of Eosin Y (EY) is induced to its single excited state (^1^EY^*^), which moves to its more stable triplet excited state (^3^EY^*^) through inter system crossing (ISC) [46–47]. ^3^EY^*^ may undergo an oxidative or reductive quenching cycle [48–50]. In this mechanism, a single electron transfer (SET) from **1** to ^3^EY* generates the amine radical cation **4**, and at the same time, ^3^EY* is reduced to the EY**^•^**^−^. In the presence of oxygen, the photoredox catalytic cycle of EY is finished via a SET oxidation, with the production of a superoxide radical anion O_2_**^•^**^−^. Deprotonation of **4** generates α-aminoalkyl radical **5**. Then **5** reacts with **2** to generate radical **6**, and the latter then undergoes cyclization to form intermediate **7**. Proton and electron transfer from **7** to O_2_**^•^**^−^ yields the final product **3** and HOO^−^. The HOO^−^ will be subsequently protonated to yield H_2_O_2_ as the by-product. H_2_O_2_ was detected after the reaction was completed by using KI/starch indicator (see the Supporting Information File 1). The involvement of radical pathway was supported by experimental result that the reaction was suppressed in the presence of TEMPO.

## Conclusion

In conclusion, we report an efficient metal-free method for the synthesis of corresponding tetrahydroquinolines from *N*,*N*-dimethylanilines and maleimides using molecular oxygen as oxidant and Eosin Y as catalyst under the irradiation of visible light. The protocol is significantly green because it utilizes visible light and atmospheric oxygen as the greenest reagents, and metal-free, cheap Eosin Y with a relatively low loading as the photocatalyst to deliver the product at room temperature in a simple one-pot procedure. This methodology expands the range of substrates in the area of visible light photoredox reactions.

## Supporting Information

File 1 Experimental section and characterization of the synthesized compounds.
