# Supplementary material for: Eosin Y-catalyzed visible-light-mediated aerobic oxidative cyclization of N,N-dimethylanilines with maleimides
Source: Beilstein J Org Chem. 2015 Apr 1;11:425–30. doi: 10.3762/bjoc.11.48 (PMC4419562; doi:10.3762/bjoc.11.48)

Supporting Information

for

Eosin Y catalyzed visible-light-mediated aerobic oxidative cyclization of *N*,*N*-dimethylanilines with maleimides

Zhongwei Liang[1,2], Song Xu[1,2,3], Wenyan Tian[1,2] and Ronghua Zhang[*1,2]

Address: [1]Department of Chemistry, Tongji University, Siping Road 1239, Shanghai 200092, China, [2]Key Laboratory of Yangtze River Water Environment, Ministry of Education, Siping Road 1239, Shanghai 200092, China and [3]College of Biological, Chemical Sciences and Engineering, Jiaxing University, Jiahang Road 118, Zhejiang 314001, China

Email: Ronghua Zhang[*] - rhzhang@tongji.edu.cn

*Corresponding author

Experimental section and characterization of the synthesized compounds

Table of contents

General remarks S2

General procedure for the reactions of *N*,*N*-dimethylanilines with maleimides S2

Photo of experimental setup S3

Test for the production of hydrogen peroxide S3

Characterization of products S4

^{1}H and ^{13}C NMR spectra of products S11

General remarks

N-Arylmaleimides were prepared according to literatures described procedures[1,2] and other commercially available reagents were used as received without further purification. Flash column chromatography was carried out over silica gel (200–300 mesh). TLC was performed using Silica Gel GF254 plates and was visualized by fluorescence quenching at 254 nm. ^{1}H NMR and ^{13}C NMR spectra were recorded on a Bruker ARX-400 MHz spectrometer in $CDCl_3$ using TMS as an internal reference with chemical shift values reported in ppm. All coupling constants (*J*) are reported in Hz. The high resolution mass spectra (HRMS) were measured on a Bruker Daltonics micrOTOF II spectrometer using ESI. Melting points were measured on a WRS-2A melting point apparatus. Two 9 W blue LEDs were used as a visible light source.

General procedure for the reactions of *N*,*N*-dimethylanilines with maleimides

To a 10 mL round bottom flask equipped with magnetic stirring bar was added *N*,*N*-dimethylaniline (**1**, 0.5 mmol, 2.0 equiv), maleimide (**2**, 0.25 mmol, 1.0 equiv), Eosin Y (water soluble, 0.03 equiv), and MeCN (3 mL). The solution was irradiated with two 9 W blue LEDs (distance app. 5 cm) at room temperature in an air atmosphere. After the completion of the reaction (indicated by TLC), the solvent was removed under reduced pressure. The residue was purified by flash column chromatography on silica gel (petroleum ether/ethyl acetate 15:1–10:1) to give the product **3**.

Photo of experimental setup

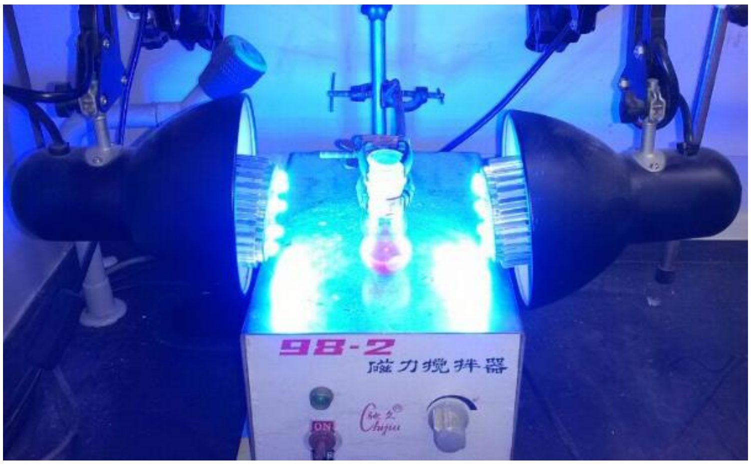

Test for the production of hydrogen peroxide

Hydrogen peroxide (H_2O_2) was detected after the reaction was completed by using KI/starch indicator.

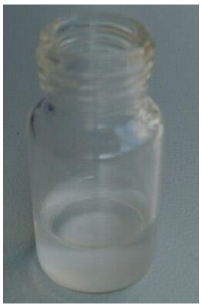 **(1)**	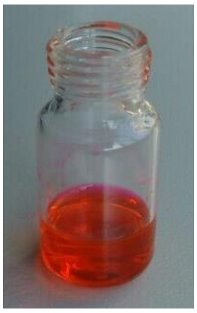 **(2)**	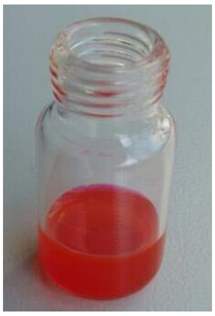 **(3)**	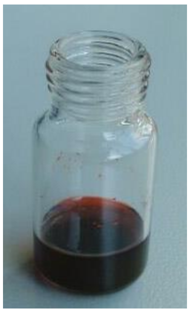 **(4)**	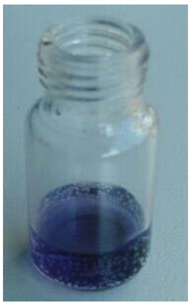 **(5)**
KI/starch indicator	Addition of reaction mixture[a] at t = 0 to **(1)**	Reaction mixture[a] at t = 18 h	Addition of reaction mixture[a] at t = 18 h to **(1)**	Addition of 30% H_2O_2 to **(1)**
Ivory	Bright red	Red	Puce	Blue

[a]Reaction mixture of 0.5 mmol of **1a** and 0.25 mmol of **2a** in 3 mL of MeCN under optimized conditions.

Characterization of products

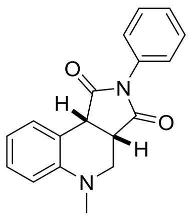

(3a*S,9b*R**)-5-Methyl-2-phenyl-3a,4,5,9b-tetrahydro-1*H*-pyrrolo[3,4-*c*]quinoline -1,3(2*H*)-dione (3a)**[3,4]

White solid; m.p.: 183-185°C.

^{1}H NMR (400 MHz, $CDCl_3$): δ 7.52 (d, *J* = 7.4 Hz, 1H), 7.42 (t, *J* = 7.5 Hz, 2H), 7.35 (t, *J* = 7.3 Hz, 1H), 7.29-7.20 (m, 3H), 6.90 (td, *J* = 7.5, 1.1 Hz, 1H), 6.74 (d, *J* = 8.2 Hz, 1H), 4.15 (d, *J* = 9.5 Hz, 1H), 3.60 (dd, *J* = 11.5, 2.7 Hz, 1H), 3.52 (ddd, *J* = 9.5, 4.4, 2.7 Hz, 1H), 3.11 (dd, *J* = 11.5, 4.4 Hz, 1H), 2.83 (s, 3H).

^{13}C NMR (101 MHz, $CDCl_3$): δ 177.7, 175.7, 148.5, 131.9, 130.3, 128.9, 128.6, 128.4, 126.3, 119.6, 118.5, 112.5, 50.6, 43.5, 42.1, 39.4.

HRMS (ESI): calcd for $C_{18}H_{16}N_2O_2$ + Na = 315.1109, found: 315.1099.

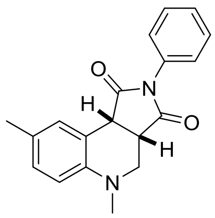

(3a*S,9b*R**)-5,8-Dimethyl-2-phenyl-3a,4,5,9b-tetrahydro-1*H*-pyrrolo[3,4-*c*]qui-noline-1,3(2*H*)-dione (3b)**[3,5]

White solid; m.p.: 187-189°C.

^{1}H NMR (400 MHz, $CDCl_3$): δ 7.41 (t, *J* = 7.4 Hz, 2H), 7.35 (d, *J* = 9.2 Hz, 2H), 7.26 (d, *J* = 7.2 Hz, 2H), 7.03 (d, *J* = 8.0 Hz, 1H), 6.64 (d, *J* = 8.3 Hz, 1H), 4.09 (d, *J* = 9.5 Hz, 1H), 3.57 (dd, *J* = 11.4, 2.7 Hz, 1H), 3.53-3.46 (m, 1H), 3.03 (dd, *J* = 11.4, 4.4 Hz, 1H), 2.79 (s, 3H), 2.30 (s, 3H).

^{13}C NMR (101 MHz, $CDCl_3$): δ 177.8, 175.8, 146.3, 132.0, 130.8 129.2, 128.94, 128.4, 126.3, 118.5, 112.5, 50.9, 43.5, 42.1, 39.5, 20.4.

HRMS (ESI): calcd for $C_{19}H_{18}N_2O_2$ + Na = 329.1266, found: 329.1254.

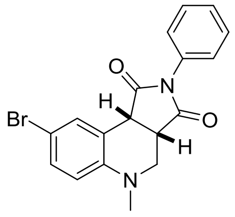

(3a*S,9b*R**)-8-Bromo-5-methyl-2-phenyl-3a,4,5,9b-tetrahydro-1*H*-pyrrolo-[3,4-*c*]quinoline-1,3(2*H*)-dione (3c)**[5]

White solid; m.p.: 156-168°C.

^{1}H NMR (400 MHz, $CDCl_3$): δ 7.63 (d, *J* = 1.7 Hz, 1H), 7.47-7.33 (m, 3H), 7.33-7.23 (m, 3H), 6.60 (d, *J* = 8.8 Hz, 1H), 4.08 (d, *J* = 9.6 Hz, 1H), 3.59 (dd, *J* = 11.5, 2.8 Hz, 1H), 3.51 (ddd, *J* = 9.6, 4.4, 2.8 Hz, 1H), 3.09 (dd, *J* = 11.6, 4.4 Hz, 1H), 2.80 (s, 3H).
^{13}C NMR (101 MHz, $CDCl_3$): δ 177.2, 175.1, 147.4, 132.6, 131.8, 131.4, 129.0, 128.6, 126.2, 120.3, 114.2, 111.6, 50.3, 43.21, 41.7, 39.4.
HRMS (ESI): calcd for $C_{18}H_{15}BrN_2O_2$ + Na = 393.0215, found: 393.0209.

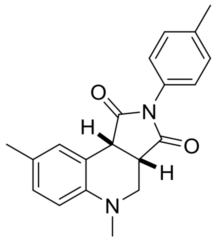

(3a*S,9b*R**)-5,8-Dimethyl-2-(*p*-tolyl)-3a,4,5,9b-tetrahydro-1*H*-pyrrolo[3,4-*c*]quinoline-1,3(2*H*)-dione (3d)[5]**
White solid; m.p.: 199-201°C.
^{1}H NMR (400 MHz, $CDCl_3$): δ 7.34 (d, *J* = 2.0 Hz, 1H), 7.27-7.18 (m, 2H), 7.13 (d, *J* = 8.3 Hz, 2H), 7.03 (dd, *J* = 8.3, 2.1 Hz, 1H), 6.65 (d, *J* = 8.3 Hz, 1H), 4.10 (d, *J* = 9.5 Hz, 1H), 3.57 (dd, *J* = 11.4, 2.7 Hz, 1H), 3.50 (ddd, *J* = 9.5, 4.4, 2.7 Hz, 1H), 3.04 (dd, *J* = 11.4, 4.4 Hz, 1H), 2.80 (s, 3H), 2.35 (s, 3H), 2.30 (s, 3H).
^{13}C NMR (101 MHz, $CDCl_3$): δ 177.9, 175.9, 146.3, 138.5, 130.8, 129.6, 129.2, 128.9, 126.1, 118.5, 112.5, 50.9, 43.5, 42.1, 39.53, 21.2, 20.42.
HRMS (ESI): calcd for $C_{20}H_{20}N_2O_2$ + Na = 343.1422, found: 343.1417.

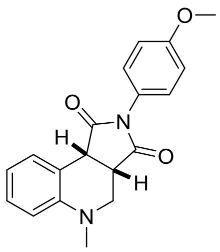

(new compound)

(3a*S,9b*R**)-2-(4-Methoxyphenyl)-5-methyl-3a,4,5,9b-tetrahydro-1*H*-pyrrolo-[3,4-*c*]quinoline-1,3(2*H*)-dione (3e)**
White solid; m.p.: 192-194°C.
^{1}H NMR (400 MHz, $CDCl_3$): δ 7.51 (d, *J* = 7.2 Hz, 1H), 7.20 (dd, *J* = 29.7, 9.1 Hz, 3H), 6.95-6.87 (m, 3H), 6.74 (d, *J* = 8.1 Hz, 1H), 4.13 (d, *J* = 9.6 Hz, 1H), 3.79 (s, 3H), 3.59 (dd, *J* = 11.5, 2.7 Hz, 1H), 3.50 (ddd, *J* = 9.6, 4.4, 2.7 Hz, 1H), 3.10 (dd, *J* = 11.5, 4.4 Hz, 1H), 2.82 (s, 3H).
^{13}C NMR (101 MHz, $CDCl_3$): δ 177.9, 176.0, 159.3, 148.4, 130.3, 128.6, 127.5, 124.6, 119.6, 118.6, 114.2, 112.5, 55.4, 50.6, 43.4, 42.0, 39.4.
HRMS (ESI): calcd for $C_{19}H_{18}N_2O_3$ + Na = 345.1215, found: 345.1210.

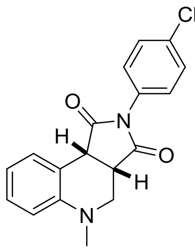

(new compound)

(3a*S,9b*R**)-2-(4-Chlorophenyl)-5-methyl-3a,4,5,9b-tetrahydro-1*H*-pyrrolo-[3,4-*c*]quinoline-1,3(2*H*)-dione (3f)**

White solid; m.p.: 191-193°C.

^{1}H NMR (400 MHz, $CDCl_3$): δ 7.51 (d, *J* = 7.5 Hz, 1H), 7.40 (d, *J* = 8.8 Hz, 2H), 7.27-7.21 (m, 3H), 6.91 (td, *J* = 7.7, 1.1 Hz, 1H), 6.75 (d, *J* = 8.2 Hz, 1H), 4.17 (d, *J* = 9.6 Hz, 1H), 3.61 (dd, *J* = 11.5, 2.6 Hz, 1H), 3.54 (ddd, *J* = 9.6, 4.3, 2.6 Hz, 1H), 3.12 (dd, *J* = 11.5, 4.4 Hz, 1H), 2.83 (s, 3H).

^{13}C NMR (101 MHz, $CDCl_3$): δ 177.4, 175.5, 148.5, 134.2, 130.4, 130.3, 129.1, 128.8, 127.5, 119.7, 118.3, 112.6, 50.6, 43.6, 42.1, 39.4.

HRMS (ESI): calcd for $C_{18}H_{15}ClN_2O_2$ + Na = 349.0720, found: 315.0721.

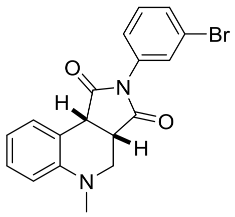

(new compound)

(3a*S,9b*R**)-2-(3-Bromophenyl)-5-methyl-3a,4,5,9b-tetrahydro-1*H*-pyrrolo-[3,4-*c*]quinoline-1,3(2*H*)-dione (3g)**

White solid; m.p.: 164-166°C.

^{1}H NMR (400 MHz, $CDCl_3$): δ 7.54-7.45 (m, 3H), 7.33-7.21 (m, 3H), 6.91 (t, *J* = 7.4 Hz, 1H), 6.75 (d, *J* = 8.1 Hz, 1H), 4.16 (d, *J* = 9.6 Hz, 1H), 3.61 (dd, *J* = 11.5, 2.6 Hz, 1H), 3.54 (ddd, *J* = 9.6, 4.3, 2.6 Hz, 1H), 3.11 (dd, *J* = 11.5, 4.3 Hz, 1H), 2.84 (s, 3H).

^{13}C NMR (101 MHz, $CDCl_3$): δ 177.3, 175.3, 148.5, 133.1, 131.6, 130.3, 130.1, 129.4, 128.8, 125.0, 122.2, 119.7, 118.3, 112.6, 50.6, 43.6, 42.1, 39.4.

HRMS (ESI): calcd for $C_{18}H_{15}BrN_2O_2$ + Na = 393.0215, found: 393.0215.

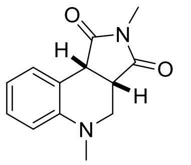

(3a*S,9b*R**)-2,5-Dimethyl-3a,4,5,9b-tetrahydro-1*H*-pyrrolo[3,4-*c*]quinoline-1,3(2*H*)-dione (3h)[3]**

White solid; m.p.: 170-172°C.

^{1}H NMR (400 MHz, $CDCl_3$): δ 7.48 (d, *J* = 7.5 Hz, 1H), 7.24-7.18 (m, 1H), 6.89 (td, *J* = 7.5, 1.1 Hz, 1H), 6.70 (d, *J* = 8.2 Hz, 1H), 4.00 (d, *J* = 9.4 Hz, 1H), 3.54 (dd, *J* = 11.5, 2.4 Hz, 1H), 3.37 (ddd, *J* = 9.5, 4.5, 2.4 Hz, 1H), 3.03 (dd, *J* = 11.5, 4.4 Hz, 1H), 2.99 (s, 3H), 2.79 (s, 3H).

^{13}C NMR (101 MHz, $CDCl_3$): δ 178.7, 176.8, 148.4, 130.2, 128.5, 119.6, 118.7, 112.4, 50.4, 43.6, 42.0, 39.4, 25.3.
HRMS (ESI): calcd for $C_{13}H_{14}N_2O_2$ + Na = 253.0953, found: 253.0947.

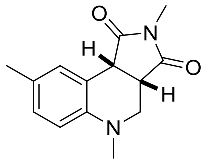

(3a*S,9b*R**)-2,5,8-Trimethyl-3a,4,5,9b-tetrahydro-1*H*-pyrrolo[3,4-*c*]quinoline-1,3(2*H*)-dione (3i)[4]**
White solid; m.p.: 178-180°C.
^{1}H NMR (400 MHz, $CDCl_3$): δ 7.29 (d, J = 2.1 Hz, 1H), 7.01 (dd, J = 8.3, 2.1 Hz, 1H), 6.60 (d, J = 8.3 Hz, 1H), 3.95 (d, J = 9.4 Hz, 1H), 3.51 (dd, J = 11.4, 2.4 Hz, 1H), 3.33 (ddd, J = 9.3, 4.3, 2.2 Hz, 1H), 3.00-2.93 (m, 4H), 2.75 (s, 3H), 2.29 (s, 3H).
^{13}C NMR (101 MHz, $CDCl_3$): δ 178.8, 176.8, 146.2, 130.6, 129.1, 128.9, 118.6, 112.4, 50.7, 43.5, 42.0, 39.5, 25.3, 20.4.
HRMS (ESI): calcd for $C_{14}H_{16}N_2O_2$ + Na = 267.1109, found: 267.1104.

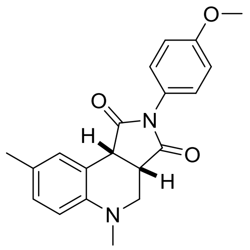

(3a*S,9b*R**)-2-(4-Methoxyphenyl)-5,8-dimethyl-3a,4,5,9b-tetrahydro-1*H*-pyrrolo[3,4-*c*]quinoline-1,3(2*H*)-dione (3j)[4]**
White solid; m.p.: 176-178°C.
^{1}H NMR (400 MHz, $CDCl_3$): δ 7.33 (d, J = 2.1 Hz, 1H), 7.16 (d, J = 9.0 Hz, 2H), 7.03 (d, J = 8.8 Hz, 1H), 6.92 (d, J = 9.0 Hz, 2H), 6.64 (d, J = 8.3 Hz, 1H), 4.07 (d, J = 9.5 Hz, 1H), 3.78 (s, 3H), 3.56 (dd, J = 11.4, 2.7 Hz, 1H), 3.47 (ddd, J = 9.6, 4.4, 2.6 Hz, 1H), 3.02 (dd, J = 11.4, 4.4 Hz, 1H), 2.78 (s, 3H), 2.29 (s, 3H).
^{13}C NMR (101 MHz, $CDCl_3$): δ 178.0, 176.0, 159.3, 146.3, 130.7, 129.1, 128.9, 127.5, 124.6, 118.5, 114.2, 112.4, 55.4, 50.9, 43.4, 42.0, 39.5, 20.4.
HRMS (ESI): calcd for $C_{20}H_{20}N_2O_3$ + Na = 359.1372, found: 359.1366.

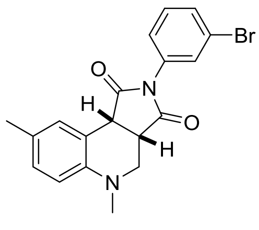

(new compound)

(3a*S,9b*R**)-2-(3-Bromophenyl)-5,8-dimethyl-3a,4,5,9b-tetrahydro-1*H*-pyrrolo-[3,4-*c*]quinoline-1,3(2*H*)-dione (3k)**
White solid; m.p.: 174-176°C.

^{1}H NMR (400 MHz, $CDCl_3$): δ 7.52-7.45 (m, 2H), 7.34-7.23 (m, 3H), 7.04 (d, *J* = 7.5 Hz, 1H), 6.65 (d, *J* = 8.3 Hz, 1H), 4.11 (d, *J* = 9.6 Hz, 1H), 3.58 (dd, *J* = 11.4, 2.6 Hz, 1H), 3.51 (ddd, *J* = 9.6, 4.3, 2.5 Hz, 1H), 3.04 (dd, *J* = 11.4, 4.3 Hz, 1H), 2.80 (s, 3H), 2.30 (s, 3H).
^{13}C NMR (101 MHz, $CDCl_3$): δ 177.3, 175.4, 146.3, 131.5, 130.7, 130.1, 129.4, 129.3, 129.0, 125.0, 122.2, 118.2, 112.6, 50.9, 43.6, 42.2, 39.5, 20.4.
HRMS (ESI): calcd for $C_{19}H_{17}BrN_2O_2$ + Na = 407.0371, found: 407.0366.

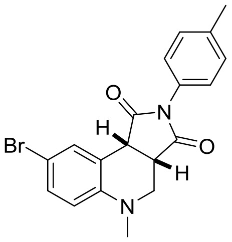

(3a*S,9b*R**)-8-Bromo-5-methyl-2-(p-tolyl)-3a,4,5,9b-tetrahydro-1*H*-pyrrolo-[3,4-*c*]quinoline-1,3(2*H*)-dione (3l)[5]**
White solid; m.p.: 193-195°C.
^{1}H NMR (400 MHz, $CDCl_3$): δ 7.62 (d, *J* = 1.5 Hz, 1H), 7.30 (dd, *J* = 8.8, 2.3 Hz, 1H), 7.26-7.20 (m, 2H), 7.12 (d, *J* = 8.4 Hz, 2H), 6.59 (d, *J* = 8.8 Hz, 1H), 4.06 (d, *J* = 9.6 Hz, 1H), 3.58 (dd, *J* = 11.5, 2.8 Hz, 1H), 3.50 (ddd, *J* = 9.6, 4.4, 2.8 Hz, 1H), 3.08 (dd, *J* = 11.5, 4.4 Hz, 1H), 2.80 (s, 3H), 2.35 (s, 3H).
^{13}C NMR (101 MHz, $CDCl_3$): δ 177.3, 175.2, 147.4, 138.6, 132.7, 131.3, 129.6, 126.0, 120.4, 114.1, 50.3, 43.2, 41.7, 39.4, 21.1.
HRMS (ESI): calcd for $C_{19}H_{17}BrN_2O_2$ + Na = 407.0371, found: 407.0366.

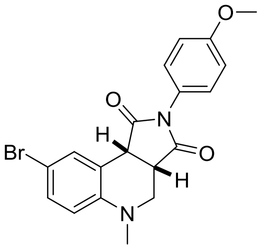

(new compound)

(3a*S,9b*R**)-8-Bromo-2-(4-methoxyphenyl)-5-methyl-3a,4,5,9b-tetrahydro-1*H*-pyrrolo[3,4-*c*]quinoline-1,3(2*H*)-dione (3m)**
White solid; m.p.: 205-207°C.
^{1}H NMR (400 MHz, $CDCl_3$): δ 7.62 (d, *J* = 1.7 Hz, 1H), 7.30 (dd, *J* = 8.8, 2.4 Hz, 1H), 7.16 (d, *J* = 9.0 Hz, 2H), 6.93 (d, *J* = 9.0 Hz, 2H), 6.59 (d, *J* = 8.8 Hz, 1H), 4.06 (d, *J* = 9.6 Hz, 1H), 3.79 (s, 3H), 3.58 (dd, *J* = 11.6, 2.8 Hz, 1H), 3.49 (ddd, *J* = 9.6, 4.4, 2.8 Hz, 1H), 3.08 (dd, *J* = 11.5, 4.4 Hz, 1H), 2.80 (s, 3H).
^{13}C NMR (101 MHz, $CDCl_3$): δ 177.4, 175.3, 159.4, 147.4, 132.7, 131.4, 127.5, 120.4, 114.3, 114.2, 111.6, 55.5, 50.4, 43.2, 41.7, 39.4.
HRMS (ESI): calcd for $C_{19}H_{17}BrN_2O_3$ + Na = 423.0320, found: 423.0315.

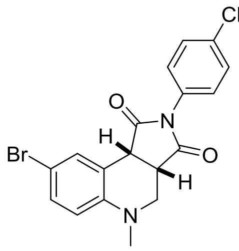 (new compound)

(3a*S,9b*R**)-8-Bromo-2-(4-chlorophenyl)-5-methyl-3a,4,5,9b-tetrahydro-1*H*-pyrrolo[3,4-*c*]quinoline-1,3(2*H*)-dione (3n)**

White solid; m.p.: 218-220°C.

^{1}H NMR (400 MHz, $CDCl_3$): δ 7.62 (d, *J* = 1.8 Hz, 1H), 7.40 (d, *J* = 8.7 Hz, 2H), 7.31 (dd, *J* = 8.7, 2.3 Hz, 1H), 7.27-7.20 (m, 2H), 6.60 (d, *J* = 8.8 Hz, 1H), 4.09 (d, *J* = 9.6 Hz, 1H), 3.60 (dd, *J* = 11.5, 2.7 Hz, 1H), 3.52 (ddd, *J* = 9.6, 4.3, 2.7 Hz, 1H), 3.09 (dd, *J* = 11.5, 4.4 Hz, 1H), 2.81 (s, 3H).

^{13}C NMR (101 MHz, $CDCl_3$): δ 176.9, 174.8, 147.4, 134.3, 132.6, 131.5, 129.2, 127.5, 120.1, 114.3, 111.7, 50.3, 43.3, 41.7, 39.4.

HRMS (ESI): calcd for $C_{18}H_{14}BrClN_2O_2$ + Na = 426.9825, found: 426.9819.

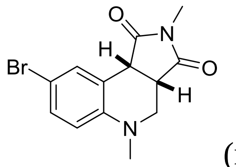 (new compound)

(3a*S,9b*R**)-8-Bromo-2,5-dimethyl-3a,4,5,9b-tetrahydro-1*H*-pyrrolo[3,4-*c*]quinoline-1,3(2*H*)-dione (3o)**

White solid; m.p.: 186-188°C.

^{1}H NMR (400 MHz, $CDCl_3$): δ 7.58 (d, *J* = 1.6 Hz, 1H), 7.28 (dd, *J* = 8.8, 2.3 Hz, 1H), 6.56 (d, *J* = 8.7 Hz, 1H), 3.94 (d, *J* = 9.5 Hz, 1H), 3.52 (dd, *J* = 11.6, 2.5 Hz, 1H), 3.36 (ddd, *J* = 9.5, 4.5, 2.5 Hz, 1H), 3.05-2.97 (m, 4H), 2.77 (s, 3H).

^{13}C NMR (101 MHz, $CDCl_3$): δ 178.2, 176.1, 147.4, 132.5, 131.2, 120.5, 114.1, 111.5, 50.2, 43.3, 41.6, 39.4, 25.4.

HRMS (ESI): calcd for $C_{13}H_{13}BrN_2O_2$ + Na = 331.0058, found: 331.0014.

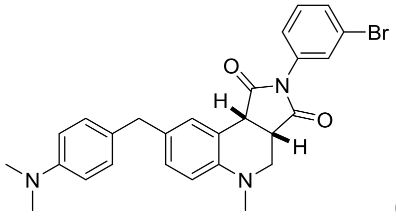 (new compound)

(3a*S,9b*R**)-2-(3-Bromophenyl)-8-(4-(dimethylamino)benzyl)-5-methyl-3a,4,5,9b-tetrahydro-1*H*-pyrrolo[3,4-*c*]quinoline-1,3(2*H*)-dione (3p)**

White solid; m.p.: 187-189°C.

^{1}H NMR (400 MHz, $CDCl_3$): δ 7.48 (d, *J* = 8.7 Hz, 2H), 7.36-7.23 (m, 4H), 7.08 (d, *J* = 8.7 Hz, 2H), 7.03 (d, *J* = 8.6 Hz, 1H), 6.67 (dd, *J* = 16.8, 8.5 Hz, 3H), 4.10 (d, *J* =

9.6 Hz, 1H), 3.84 (s, 2H), 3.56 (dd, *J* = 11.4, 2.6 Hz, 1H), 3.48 (ddd, *J* = 9.7, 4.4, 2.5 Hz, 1H), 3.03 (dd, *J* = 11.4, 4.4 Hz, 1H), 2.90 (s, 6H), 2.78 (s, 3H).
^{13}C NMR (101 MHz, $CDCl_3$): δ 177.4, 175.4, 146.7, 133.5, 133.2, 131.5, 130.5, 130.1, 129.6, 129.4, 129.0, 125.0, 122.2, 118.3, 113.0, 112.7, 50.9, 43.6, 42.2, 40.8, 40.0, 39.5.
HRMS (ESI): calcd for $C_{27}H_{26}BrN_3O_2$ + Na = 526.1106, found: 526.1101.

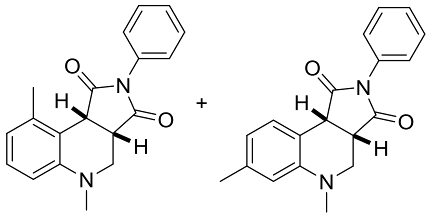

(3a*S,9b*R**)-5,9-Dimethyl-2-phenyl-3a,4,5,9b-tetrahydro-1*H*-pyrrolo[3,4-*c*]quinoline-1,3(2*H*)-dione (3q1)[5]**
(3a*S,9b*R**)-5,7-Dimethyl-2-phenyl-3a,4,5,9b-tetrahydro-1*H*-pyrrolo[3,4-*c*]quinoline-1,3(2*H*)-dione (3q2)[5]**
^{1}H NMR (400 MHz, $CDCl_3$): δ 7.44-7.37 (m, 2.4H), 7.36-7.30 (m, 1.1H), 7.28-7.21 (m, 2.1H), 7.20-7.09 (m, 1.6H), 6.87 (t, *J* = 7.3 Hz, 0.4H), 6.81 (d, *J* = 7.5 Hz, 0.6H), 6.73 (d, *J* = 7.5 Hz, 1.2H), 6.62 (d, *J* = 8.1 Hz, 0.7H), 6.55 (s, 0.3H), 4.49 (d, *J* = 9.8 Hz, 0.7H), 4.08 (d, *J* = 9.6 Hz, 0.4H), 3.60-3.53 (m, 1H), 3.52-3.44 (m, 1H), 3.05 (dd, *J* = 11.5, 4.4 Hz, 0.3H), 2.91 (dd, *J* = 11.4, 4.8 Hz, 0.7H), 2.80 (s, 1H), 2.76 (s, 2H), 2.57 (s, 2H), 2.31 (s, 1H).
^{13}C NMR (101 MHz, $CDCl_3$): δ 178.7, 178.0, 176.1, 175.7, 155.8, 150.1, 148.3, 138.5, 138.4, 131.9, 131.8, 130.0, 129.4, 128.9, 128.9, 128.5, 128.4, 127.9, 126.4, 126.3, 122.6, 120.4, 120.2, 119.4, 115.5, 115.2, 113.2, 110.6, 52.5, 50.6, 44.7, 43.4, 41.8, 39.8, 39.4, 39.3, 21.6, 20.3.
HRMS (ESI): calcd for $C_{19}H_{18}N_2O_2$ + Na = 329.1266, found: 329.1257.

References:

1. Corey, E. J.; Sarshar, S.; Lee, D. H. *J. Am. Chem. Soc.* **1994**, *116*, 12089-12090.

2. Uchoa, A. F.; de Oliveira, K. T.; Baptista, M. S.; Bortoluzzi, A. J.; Iamamoto, Y.; Serra, O. A. *J. Org. Chem.* **2011**, *76*, 8824-8832.

3. Roy, R. B.; Swan, G. A. *Chem. Commun.* **1968**, 1446-1447.

4. Nishino, M.; Hirano, K.; Satoh, T.; Miura, M. *J. Org. Chem.* **2011**, *76*, 6447-6451.

5. Ju, X.-H.; Li, D.-J.; Li, W.-F.; Yu, W.; Bian, F.-L. *Adv. Synth. Catal.* **2012**, *354*, 3561-3567.

^{1}H and ^{13}C NMR spectra of products

3a

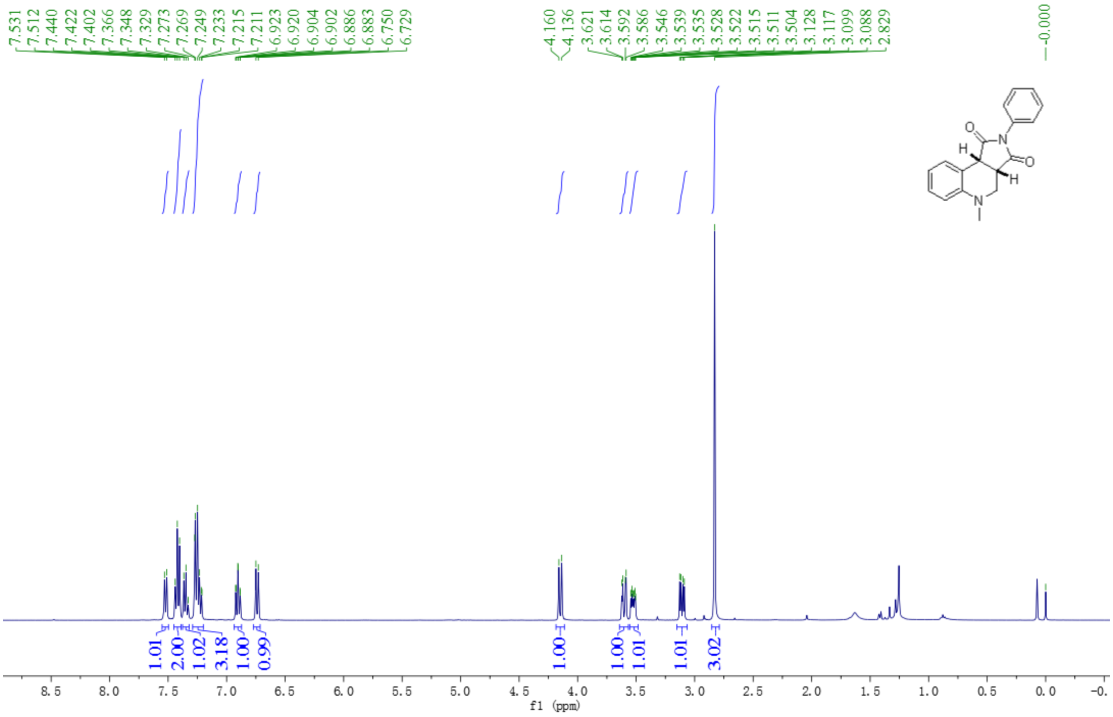

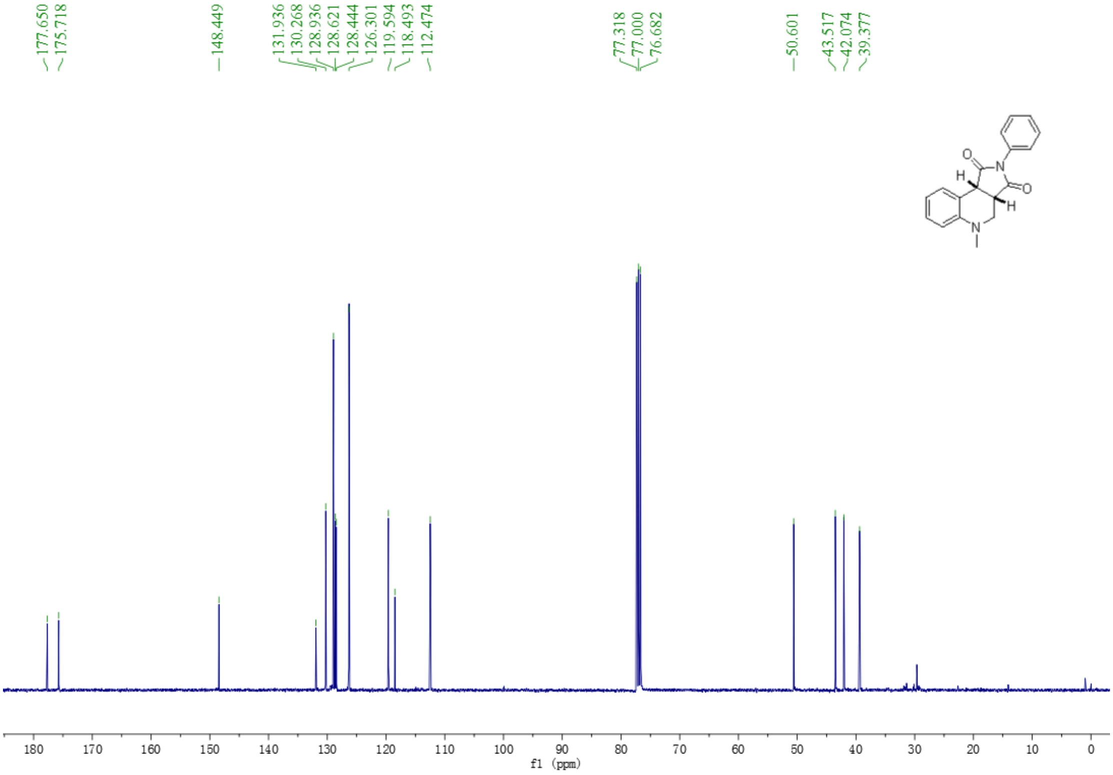

3b

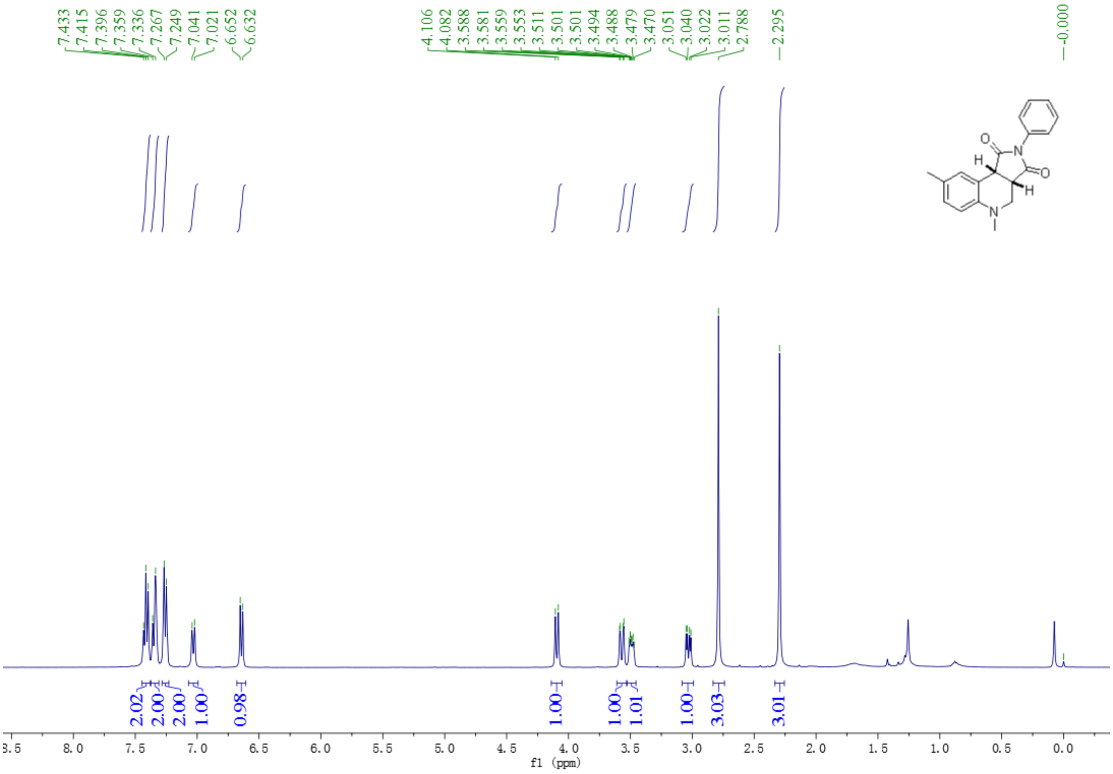

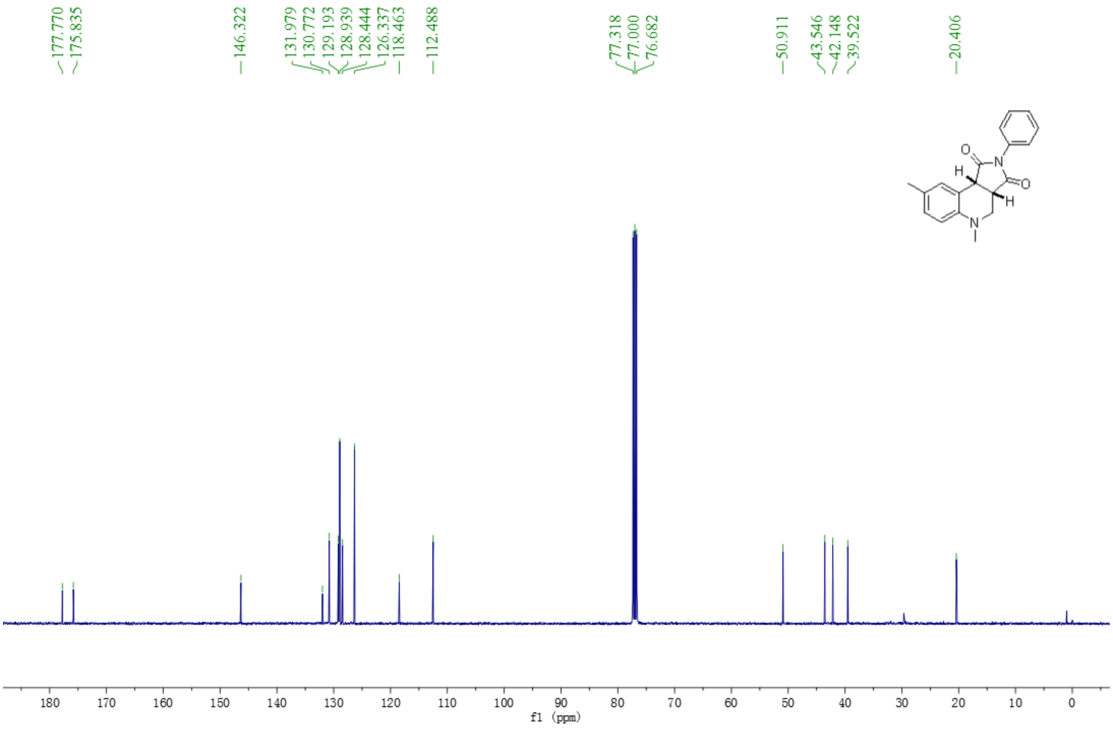

3c

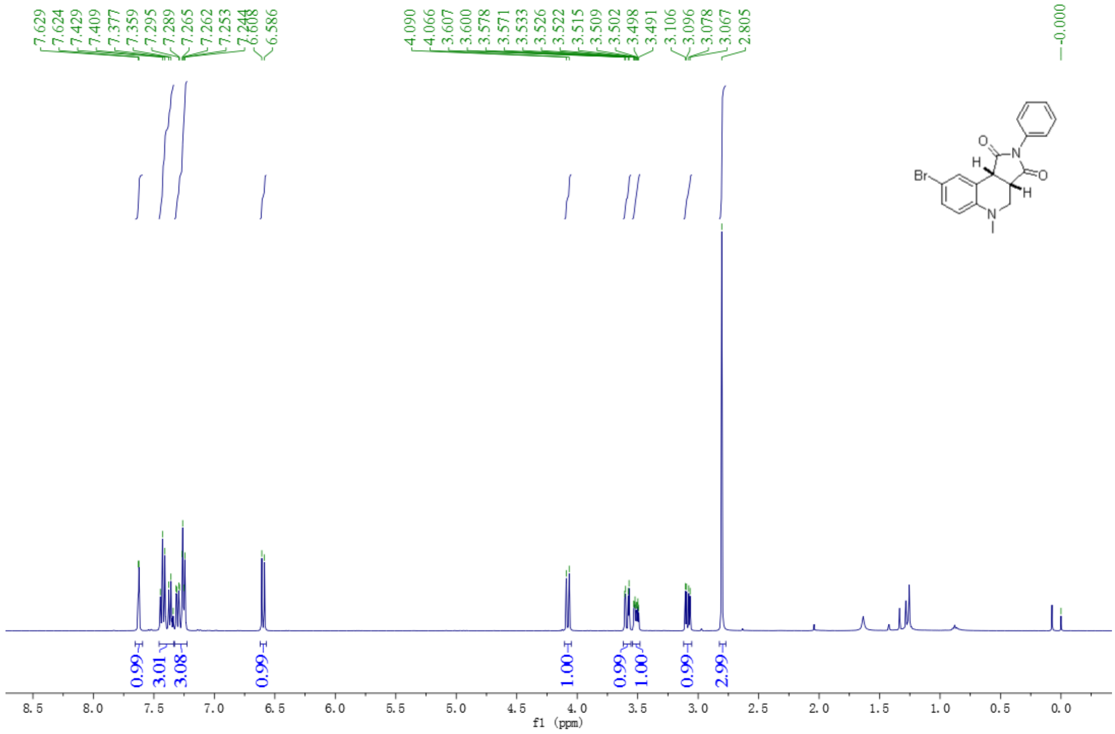
7.629
7.624
7.429
7.409
7.377
7.359
7.295
7.289
7.265
7.262
7.253
7.244
6.608
6.586
4.090
4.066
3.607
3.600
3.578
3.571
3.533
3.526
3.522
3.515
3.509
3.502
3.498
3.491
3.106
3.096
3.078
3.067
2.805
0.000
Br
O
N
H
0.99
3.01
3.08
0.99
1.00
0.99
1.00
0.99
2.99
8.5 8.0 7.5 7.0 6.5 6.0 5.5 5.0 4.5 4.0 3.5 3.0 2.5 2.0 1.5 1.0 0.5 0.0
f1 (ppm)

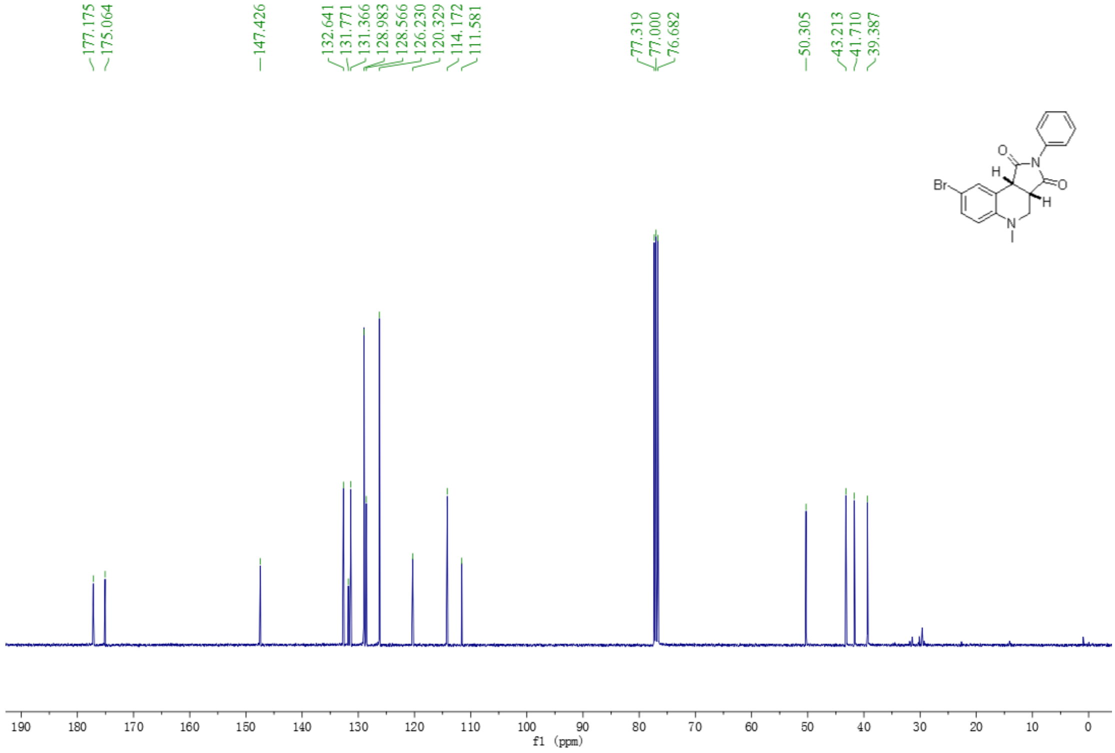
177.175
175.064
147.426
132.641
131.771
131.366
128.983
128.566
126.230
120.329
114.172
111.581
77.319
77.000
76.682
50.305
43.213
41.710
39.387
Br
O
N
H
190 180 170 160 150 140 130 120 110 100 90 80 70 60 50 40 30 20 10 0
f1 (ppm)

3d

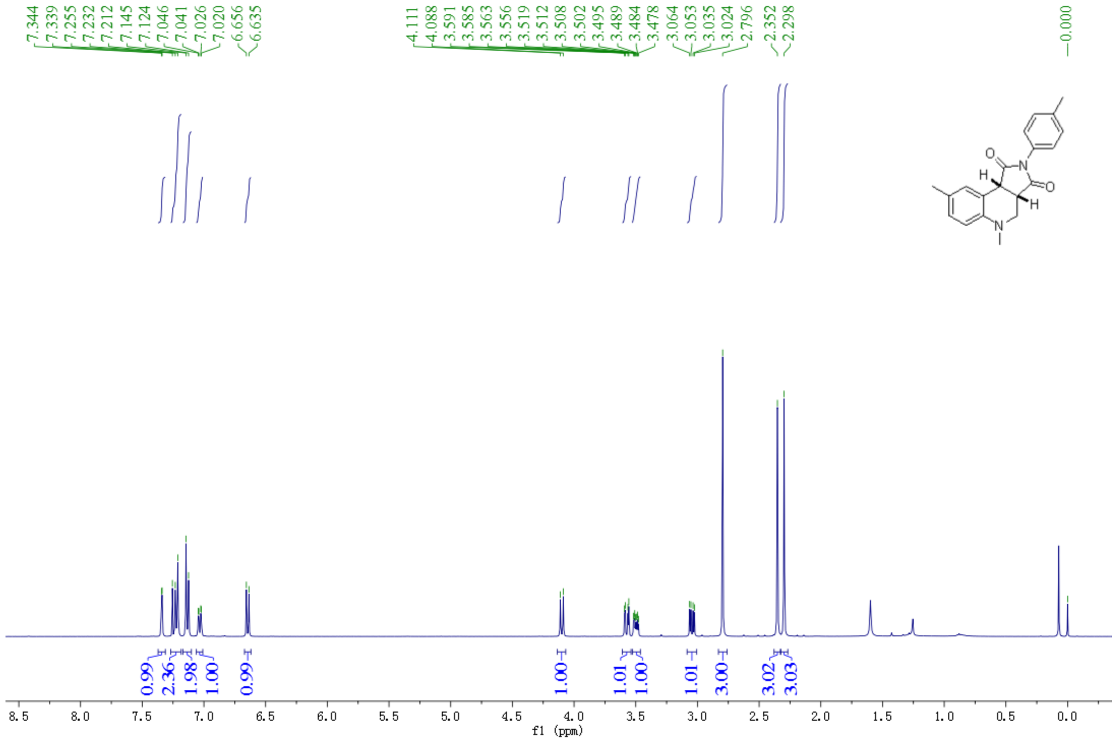
7.344
7.339
7.255
7.232
7.212
7.145
7.124
7.046
7.041
7.026
7.020
6.656
6.635
4.111
4.088
3.591
3.585
3.563
3.556
3.519
3.512
3.508
3.502
3.495
3.489
3.484
3.478
3.064
3.053
3.035
3.024
2.796
2.352
2.298
0.000
0.99
2.36
1.98
1.00
0.99
1.00
1.01
1.00
1.01
3.00
3.02
3.03
f1 (ppm)

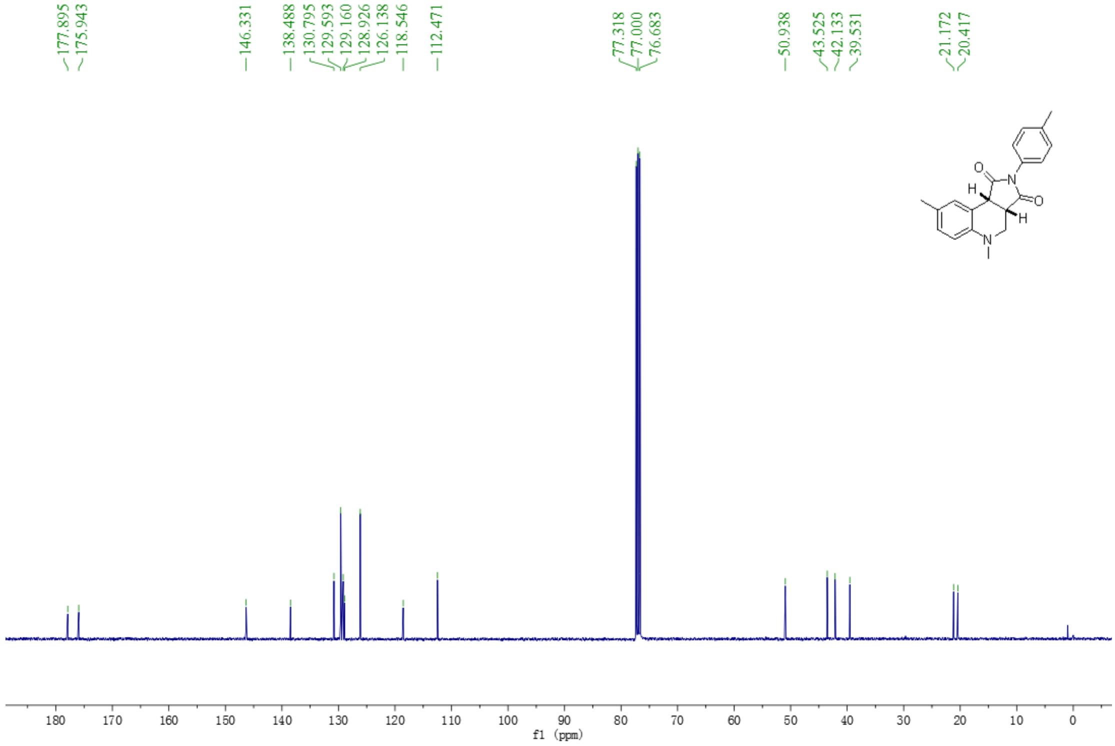
177.895
175.943
146.331
138.488
130.795
129.593
129.160
128.926
126.138
118.546
112.471
77.318
77.000
76.683
50.938
43.525
42.133
39.531
21.172
20.417
f1 (ppm)

3e

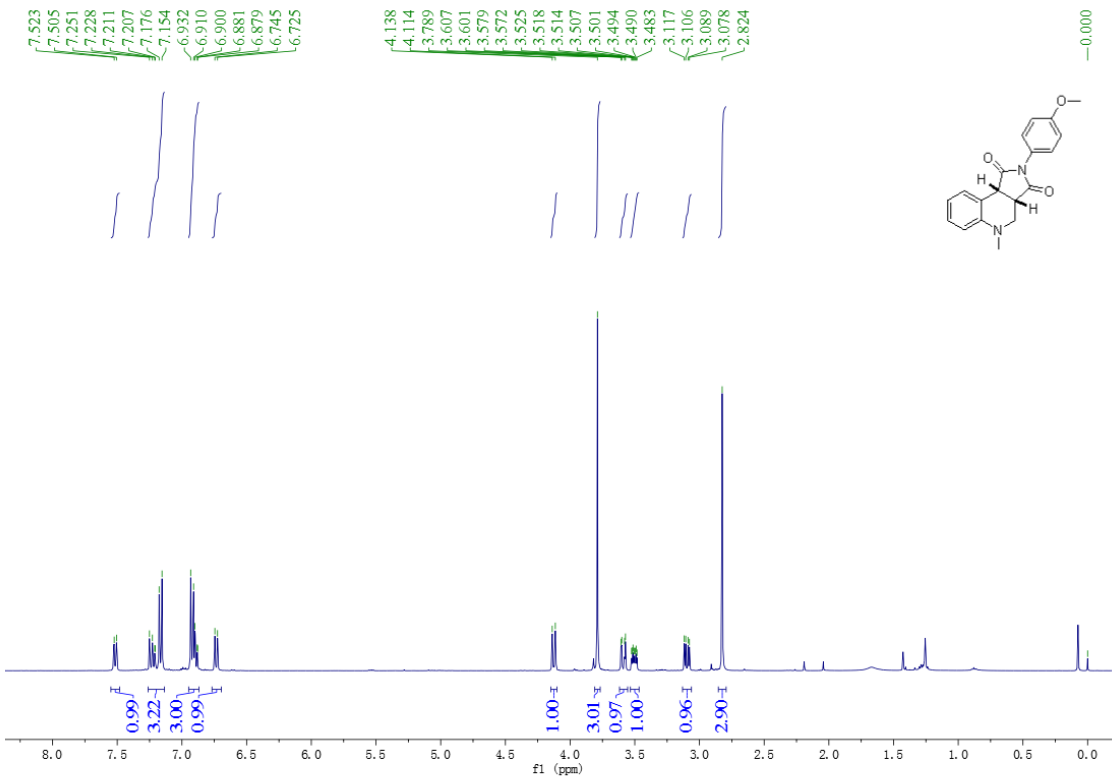

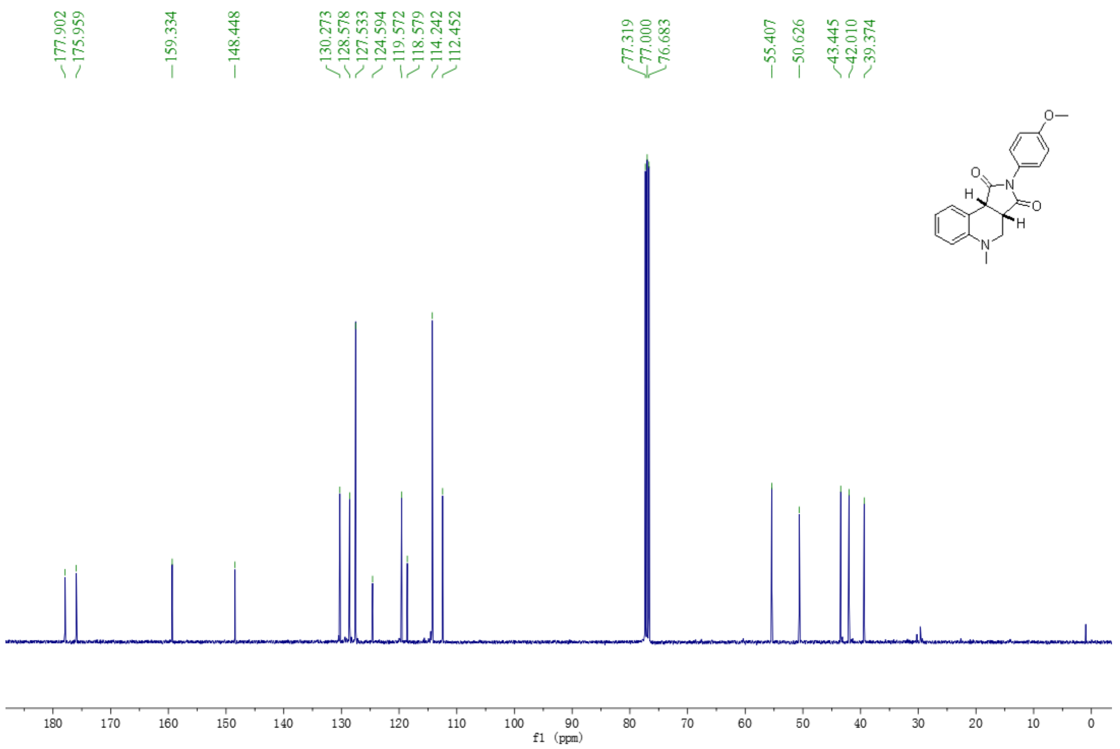

3f

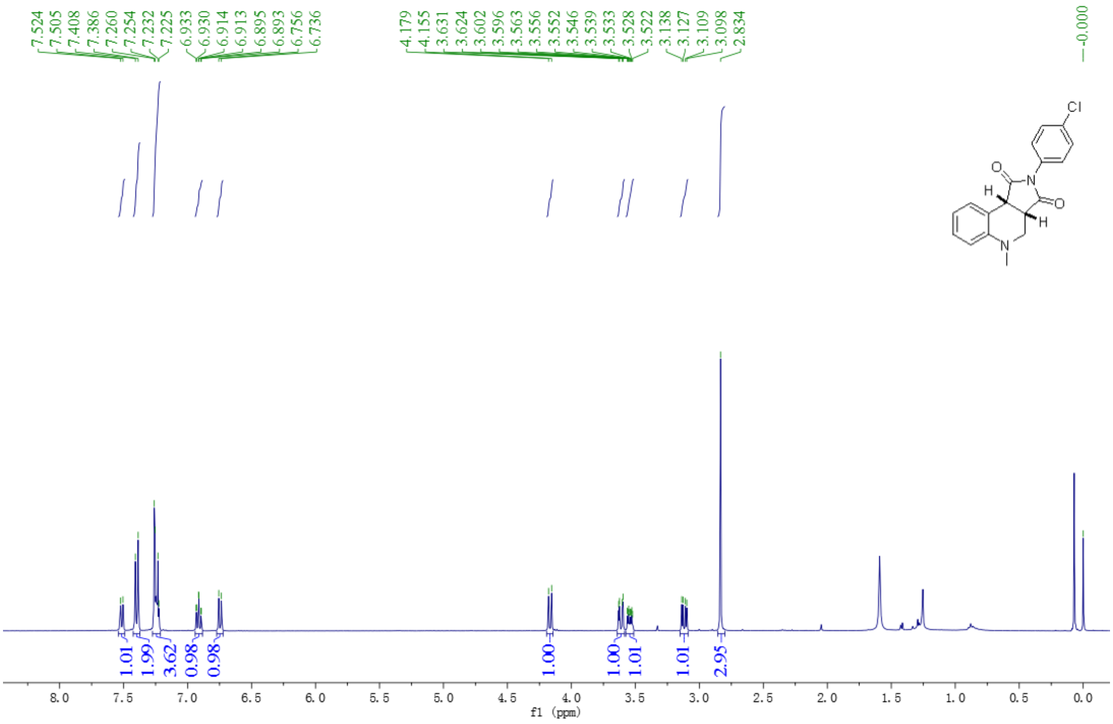

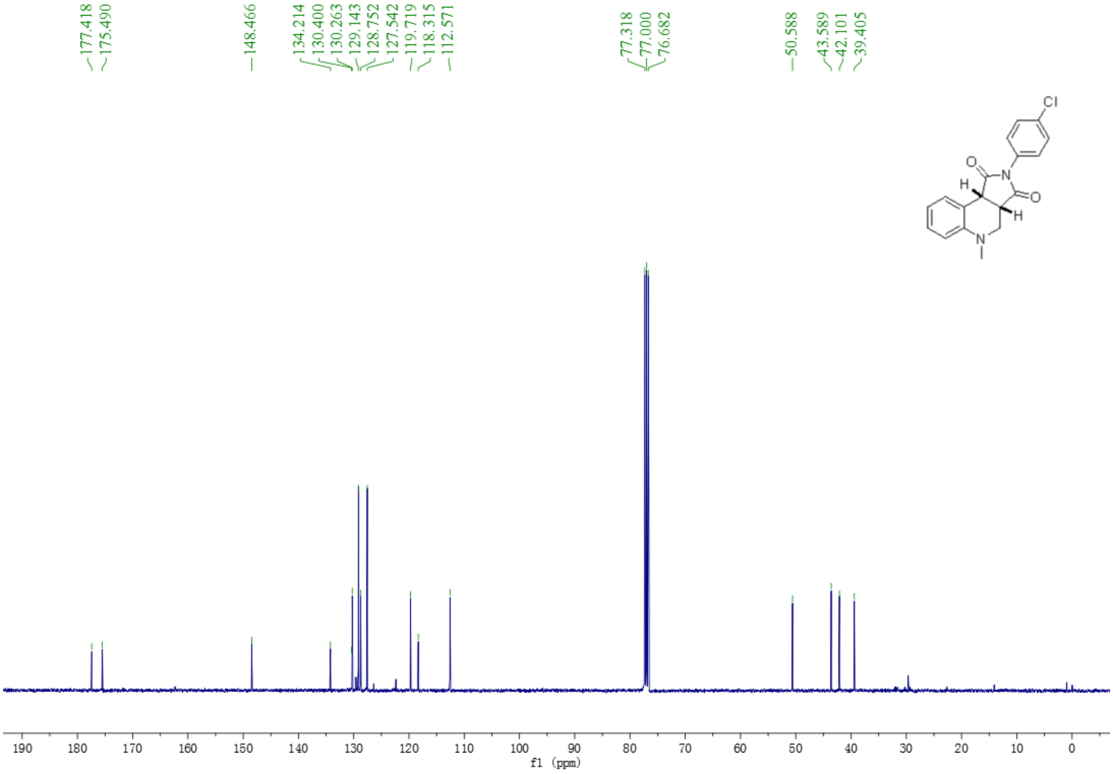

3g

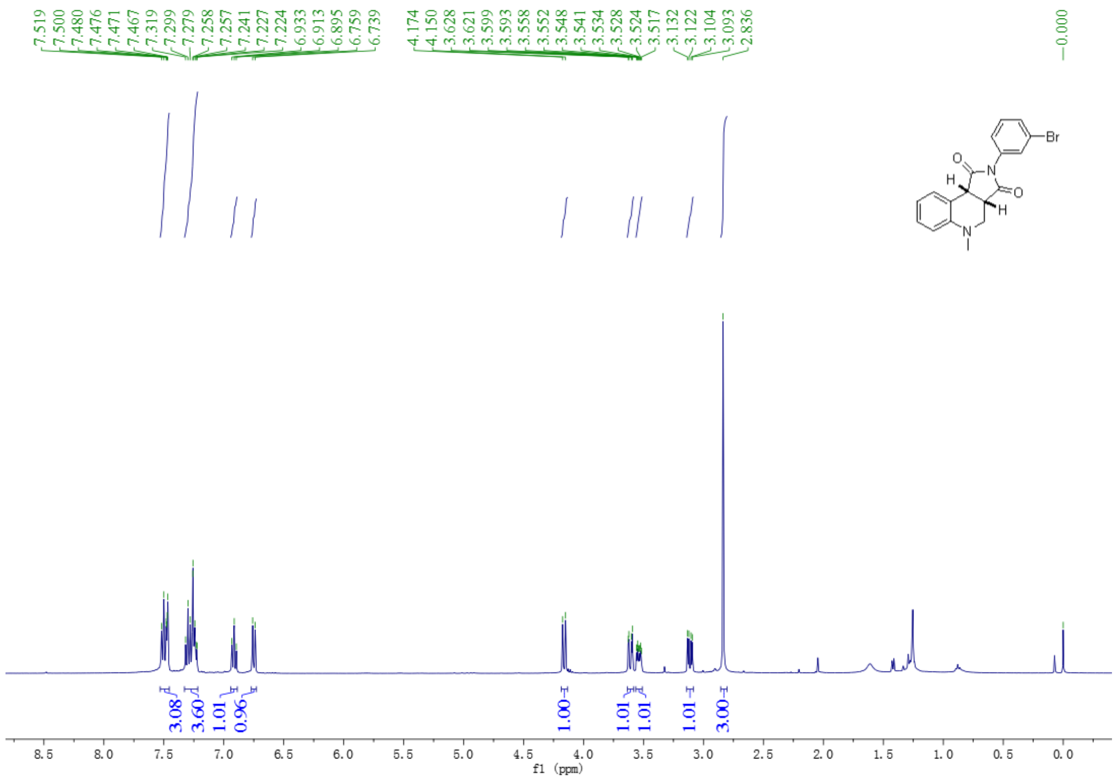

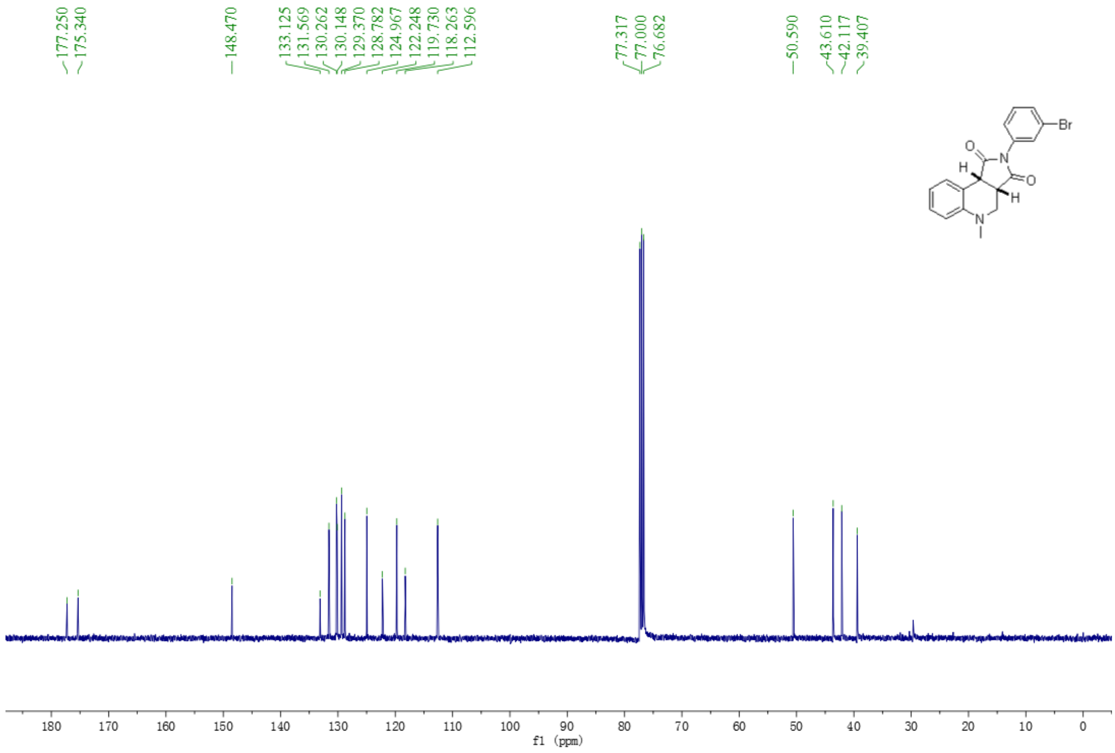

3h

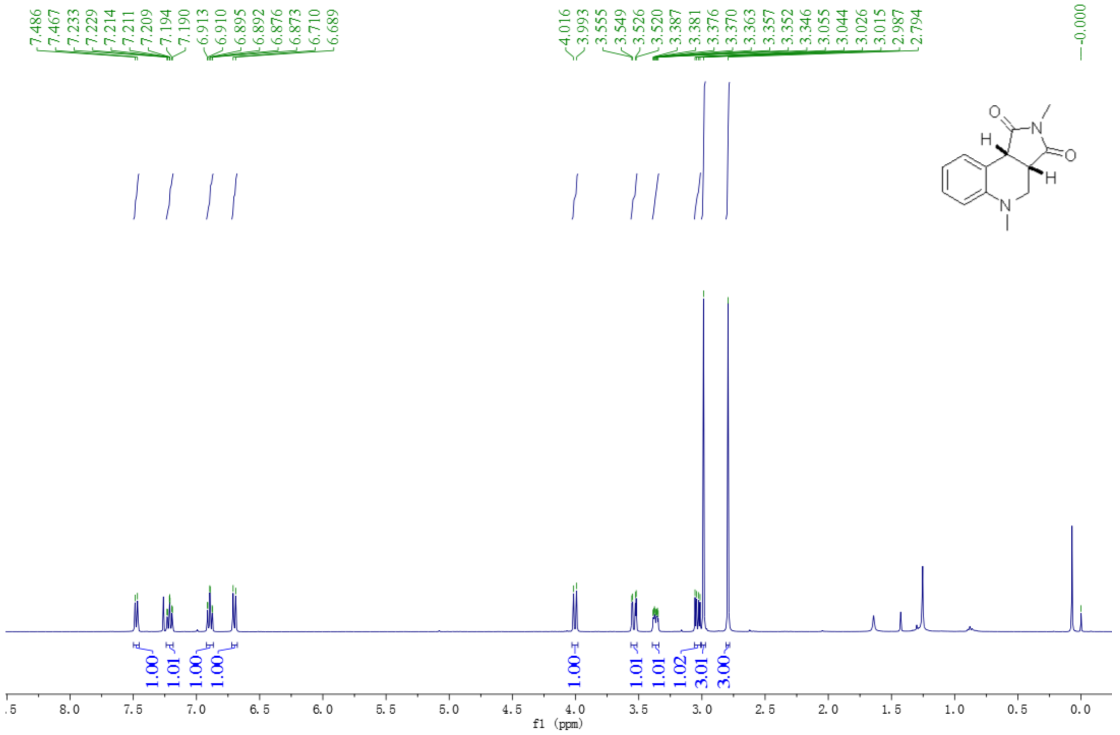
7.486
7.467
7.233
7.229
7.214
7.211
7.209
7.194
7.190
6.913
6.910
6.895
6.892
6.876
6.873
6.710
6.689
4.016
3.993
3.555
3.549
3.526
3.520
3.387
3.381
3.376
3.370
3.363
3.357
3.352
3.346
3.055
3.044
3.026
3.015
2.987
2.794
-0.000
1.00
1.01
1.00
1.00
1.00
1.01
1.01
1.02
3.01
3.00
f1 (ppm)

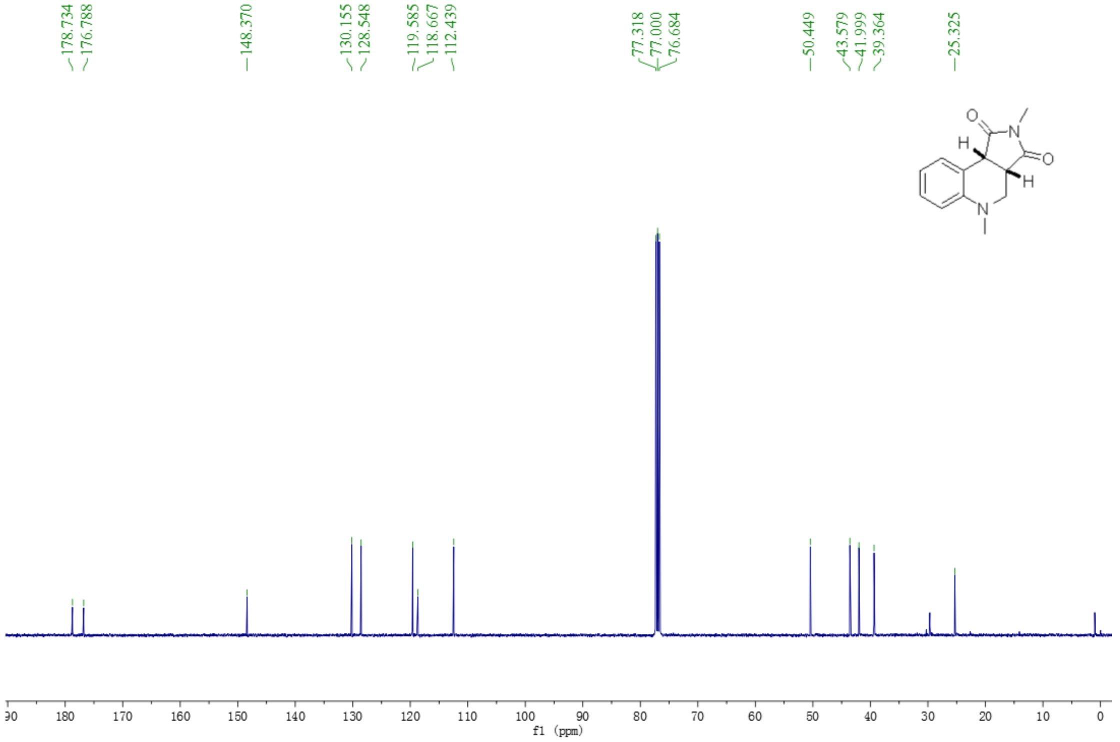
178.734
176.788
148.370
130.155
128.548
119.585
118.667
112.439
77.318
77.000
76.684
50.449
43.579
41.999
39.364
25.325
f1 (ppm)

3i

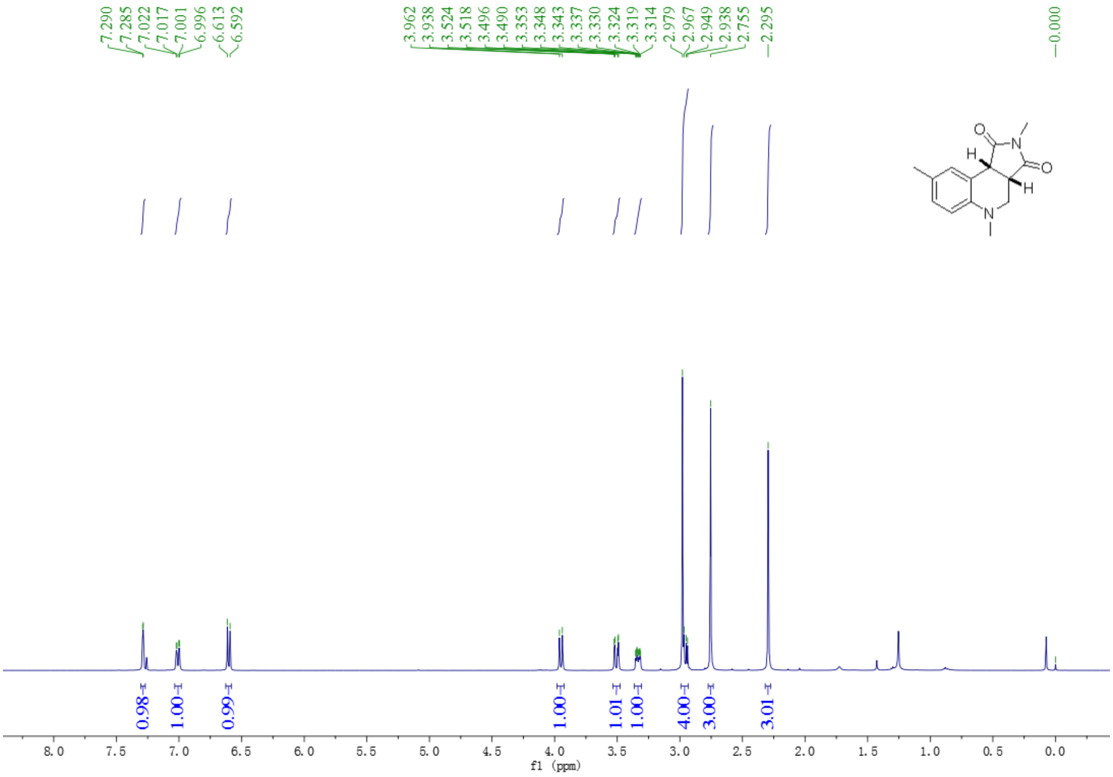

7.290
7.285
7.022
7.017
7.001
6.996
6.613
6.592
3.962
3.938
3.524
3.518
3.496
3.490
3.353
3.348
3.343
3.337
3.330
3.324
3.319
3.314
2.979
2.967
2.949
2.938
2.755
2.295
0.000
0.98
1.00
0.99
1.00
1.01
1.00
4.00
3.00
3.01
f1 (ppm)

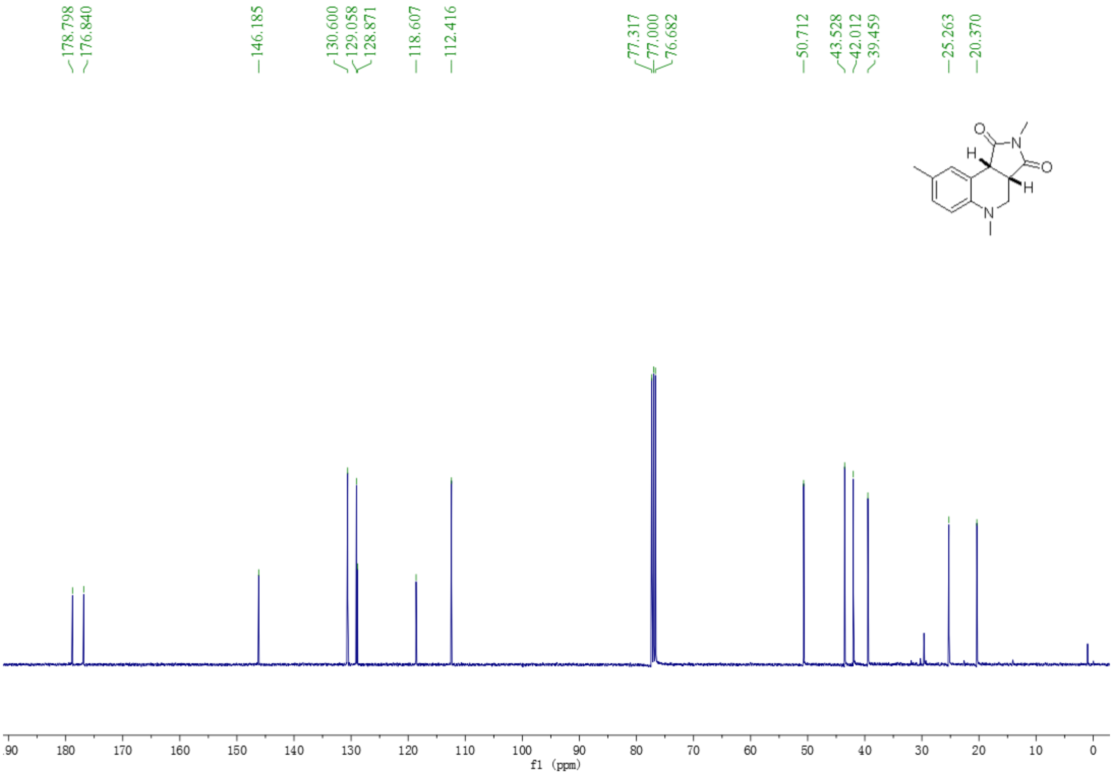

178.798
176.840
146.185
130.600
129.058
128.871
118.607
112.416
77.317
77.000
76.682
50.712
43.528
42.012
39.459
25.263
20.370
f1 (ppm)

3j

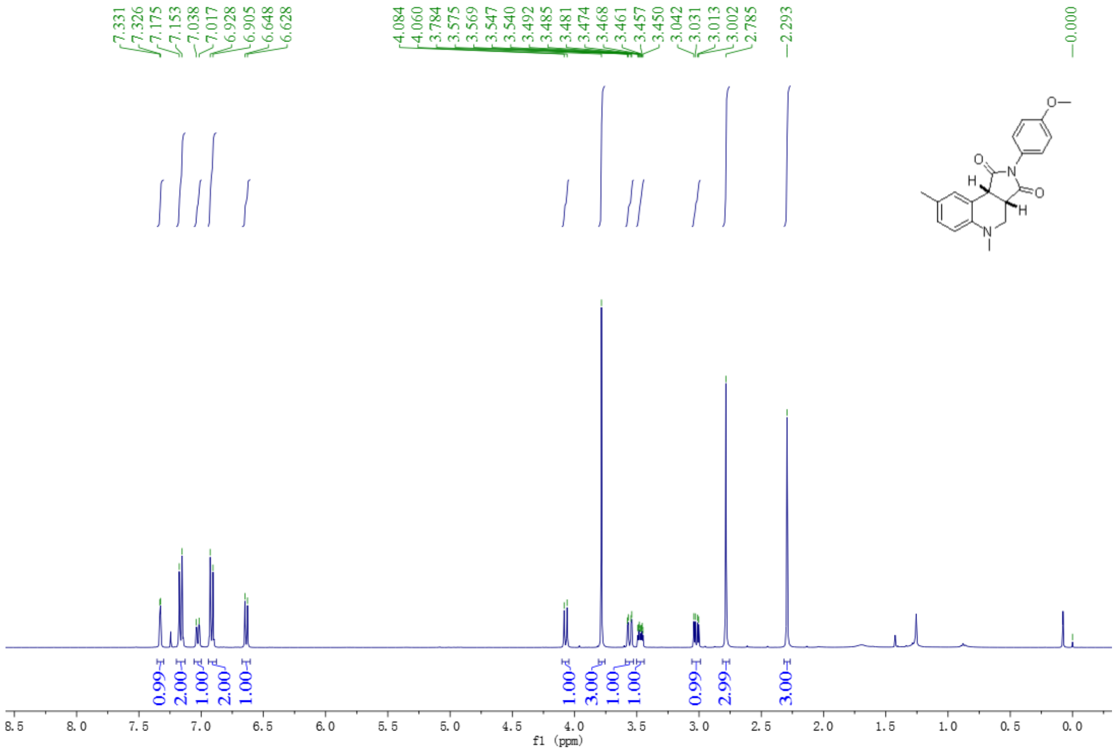

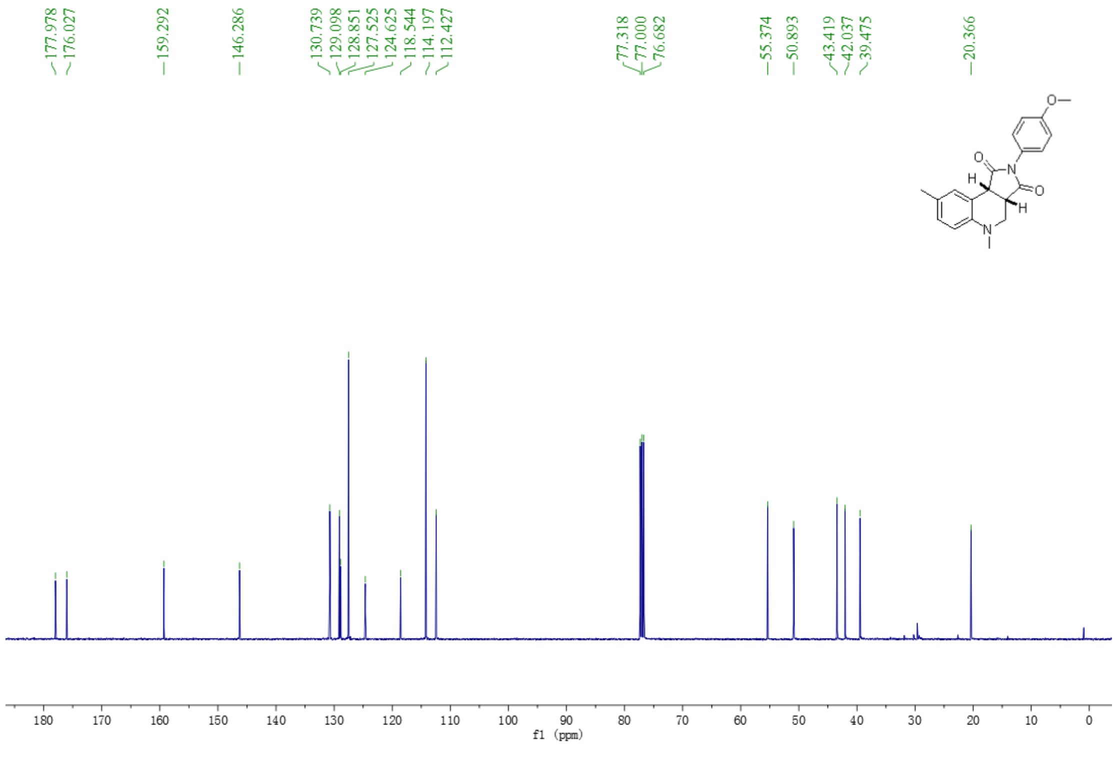

3k

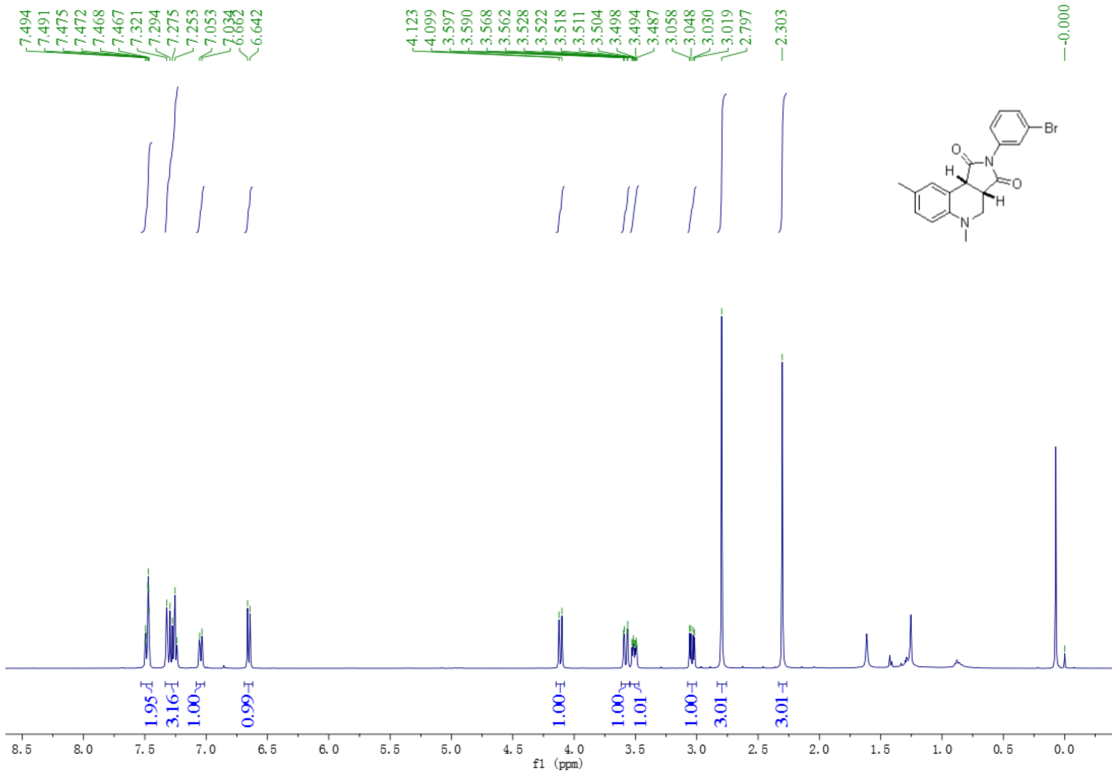
7.494
7.491
7.475
7.472
7.468
7.467
7.321
7.294
7.275
7.253
7.053
7.034
6.662
6.642
4.123
4.099
3.597
3.590
3.568
3.562
3.528
3.522
3.518
3.511
3.504
3.498
3.494
3.487
3.058
3.048
3.030
3.019
2.797
2.303
-0.000
Br
O
N
H
1.95
3.16
1.00
0.99
1.00
1.00
1.01
1.00
3.01
3.01
f1 (ppm)

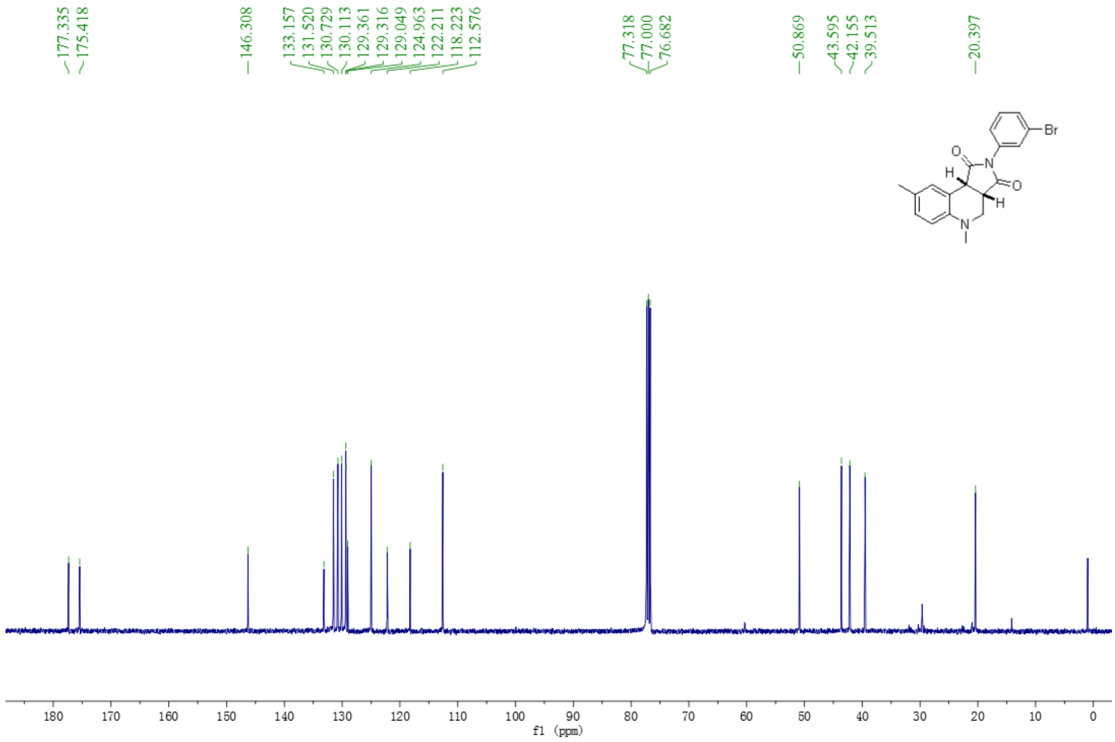
177.335
175.418
146.308
133.157
131.520
130.729
130.113
129.361
129.316
129.049
124.963
122.211
118.223
112.576
77.318
77.000
76.682
50.869
43.595
42.155
39.513
20.397
Br
O
N
H
f1 (ppm)

3l

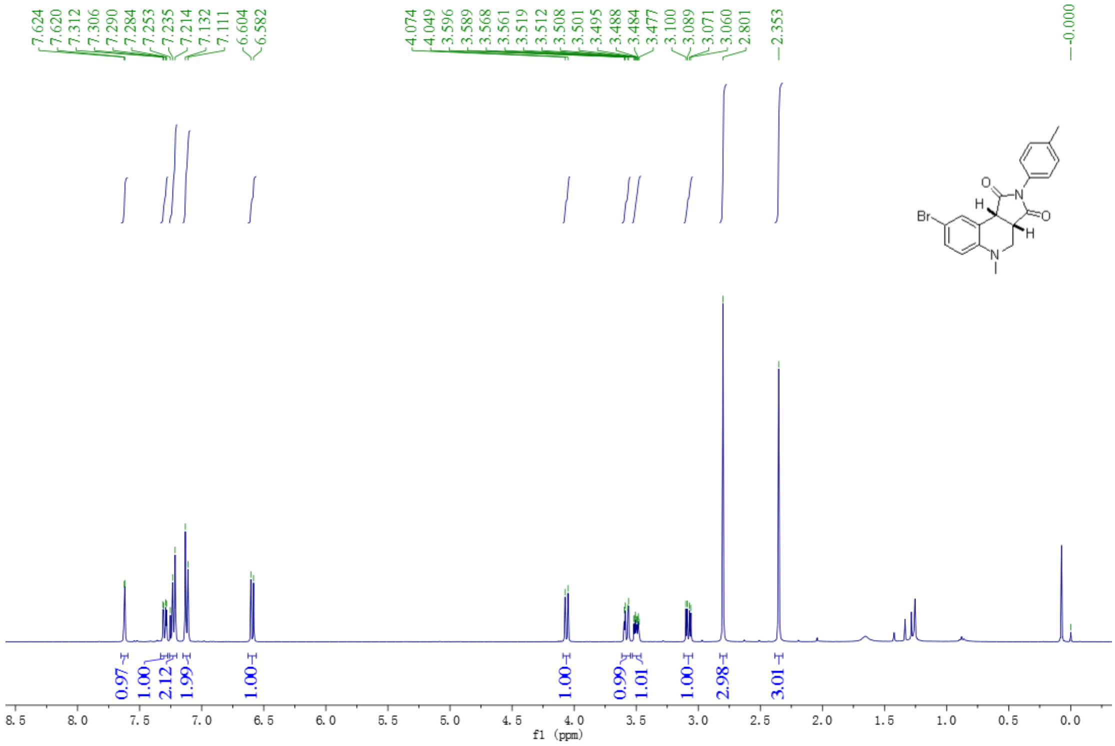

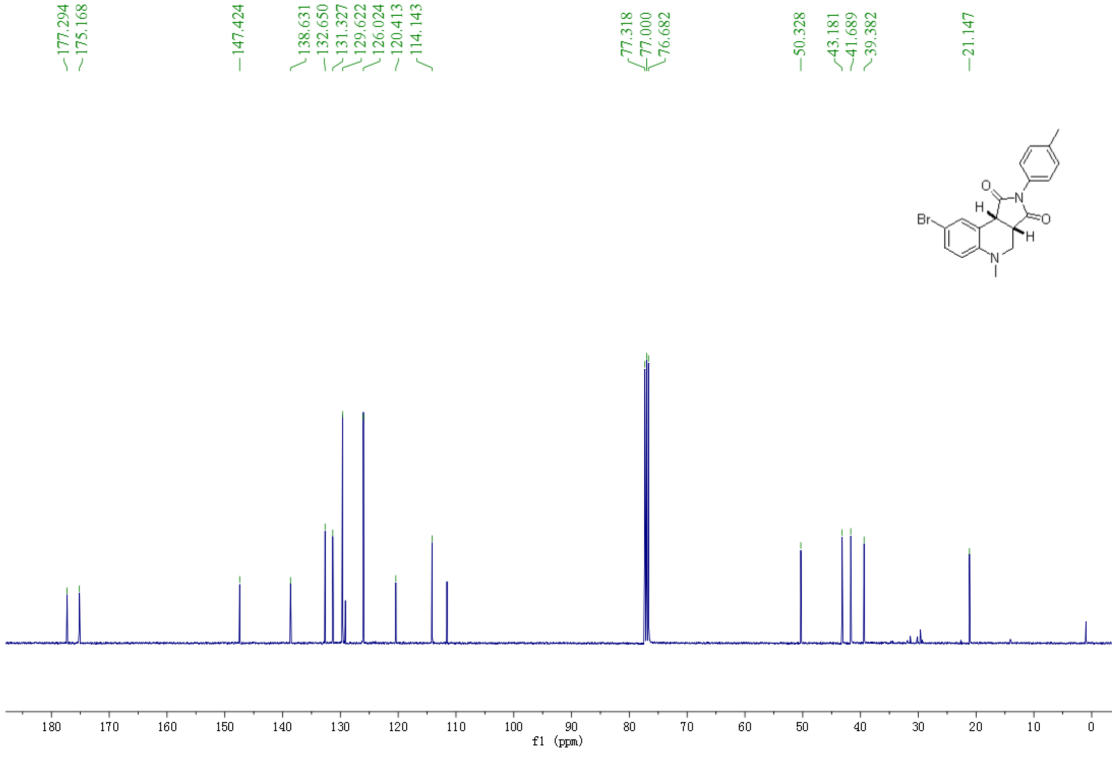

3m

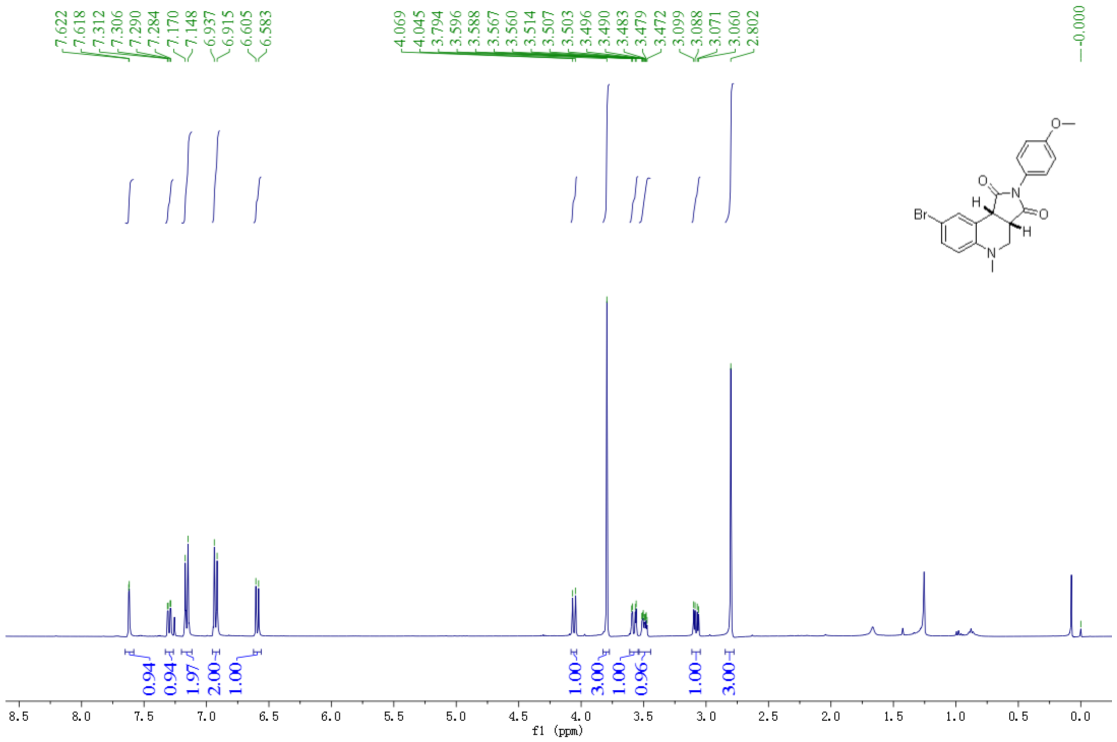
7.622
7.618
7.312
7.306
7.290
7.284
7.170
7.148
6.937
6.915
6.605
6.583
4.069
4.045
3.794
3.596
3.588
3.567
3.560
3.514
3.507
3.503
3.496
3.490
3.483
3.479
3.472
3.099
3.088
3.071
3.060
2.802
0.000
Br
H
O
N
O
H
N
0.94
0.94
1.97
2.00
1.00
1.00
3.00
1.00
0.96
1.00
3.00
f1 (ppm)

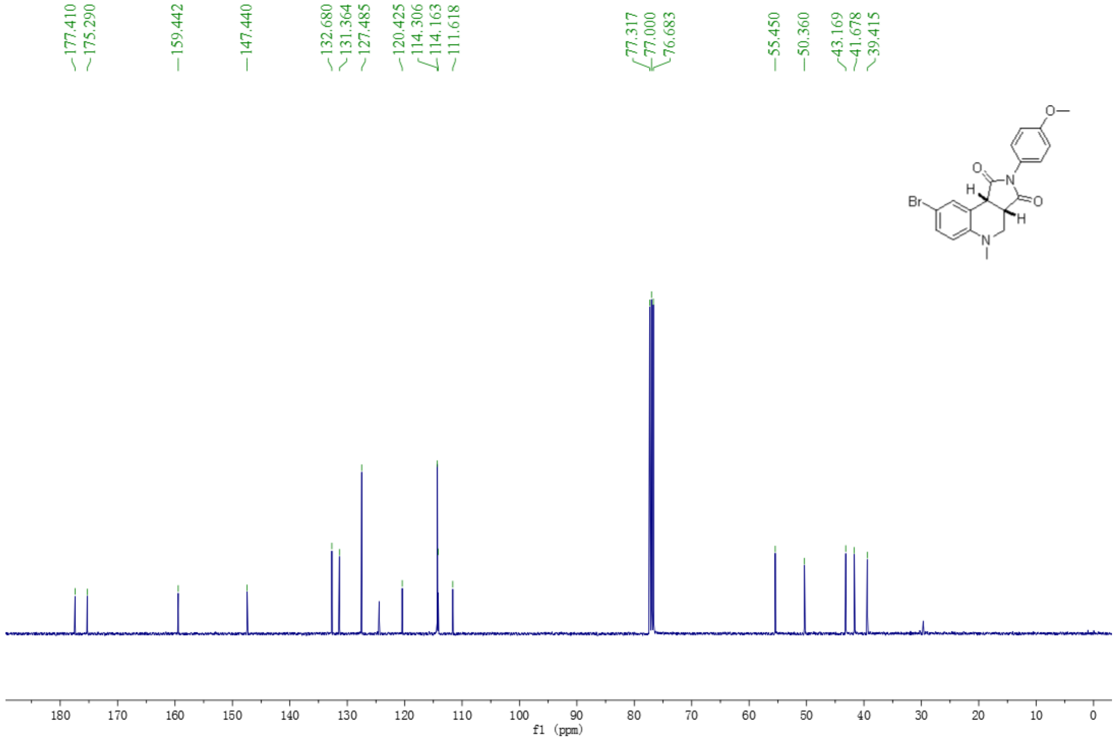
177.410
175.290
159.442
147.440
132.680
131.364
127.485
120.425
114.306
114.163
111.618
77.317
77.000
76.683
55.450
50.360
43.169
41.678
39.415
Br
H
O
N
O
H
N
f1 (ppm)

3n

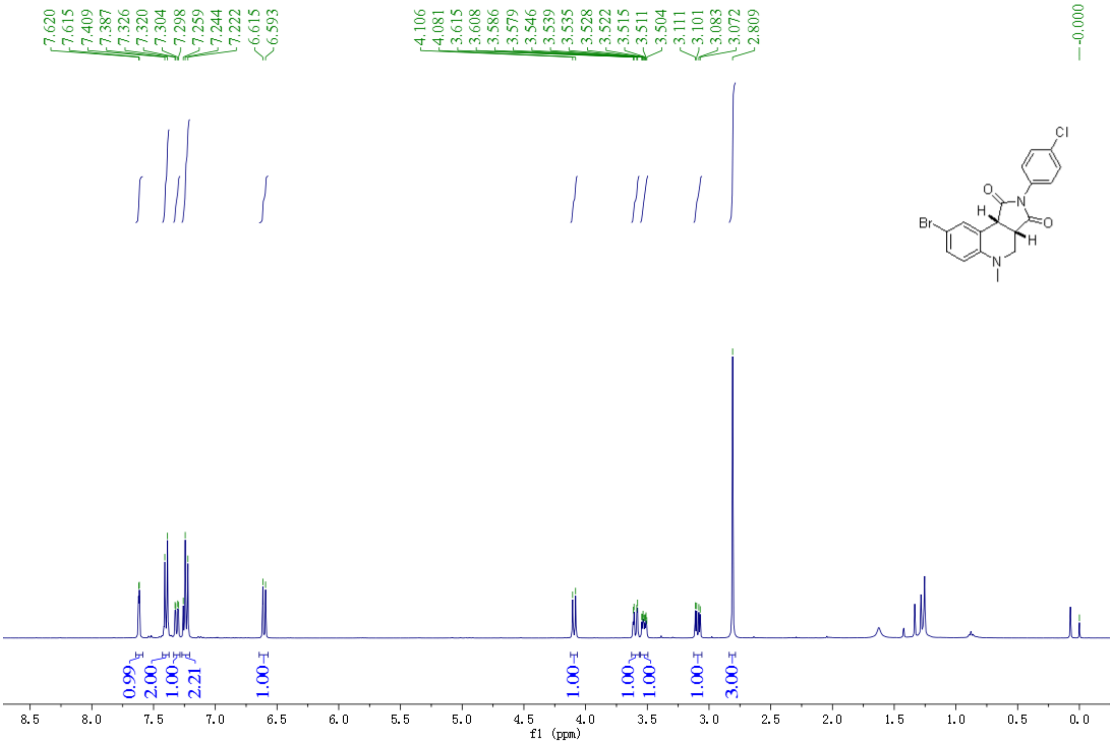
7.620
7.615
7.409
7.387
7.326
7.320
7.304
7.298
7.259
7.244
7.222
6.615
6.593
4.106
4.081
3.615
3.608
3.586
3.579
3.546
3.539
3.535
3.528
3.522
3.515
3.511
3.504
3.111
3.101
3.083
3.072
2.809
0.000
Cl
O
N
H
O
Br
H
N
0.99
2.00
1.00
2.21
1.00
1.00
1.00
1.00
1.00
3.00
8.5 8.0 7.5 7.0 6.5 6.0 5.5 5.0 4.5 4.0 3.5 3.0 2.5 2.0 1.5 1.0 0.5 0.0
f1 (ppm)

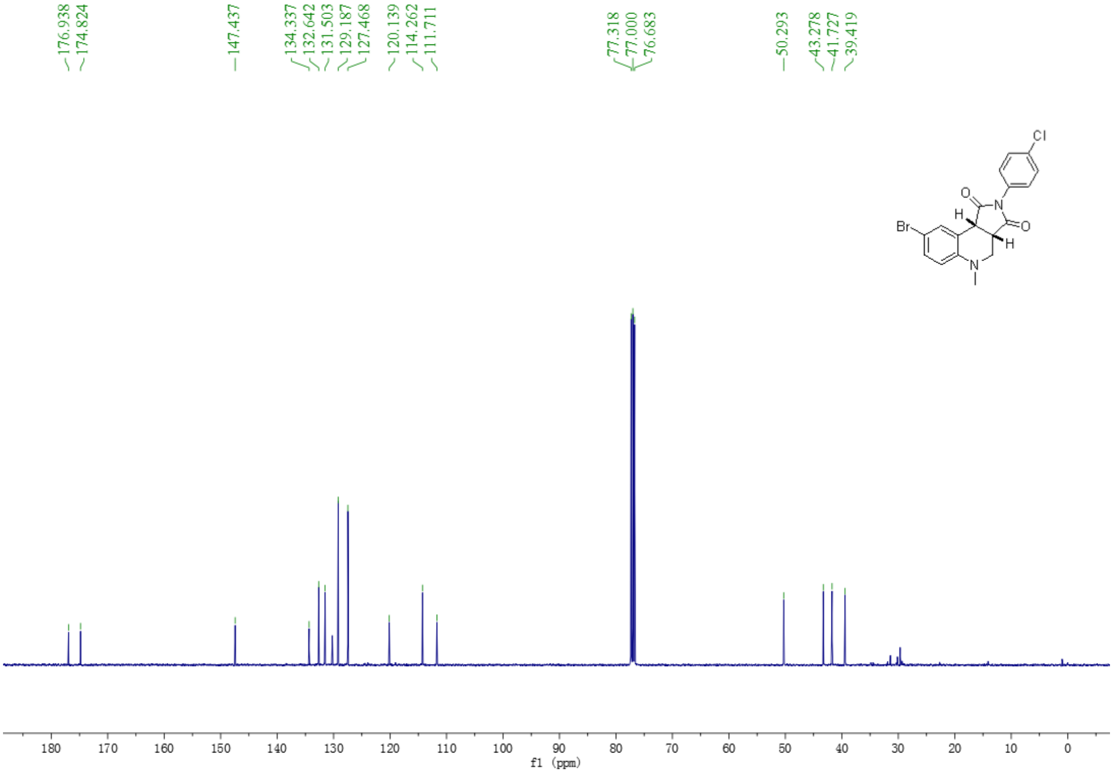
176.938
174.824
147.437
134.337
132.642
131.503
129.187
127.468
120.139
114.262
111.711
77.318
77.000
76.683
50.293
43.278
41.727
39.419
Cl
O
N
H
O
Br
H
N
180 170 160 150 140 130 120 110 100 90 80 70 60 50 40 30 20 10 0
f1 (ppm)

3o

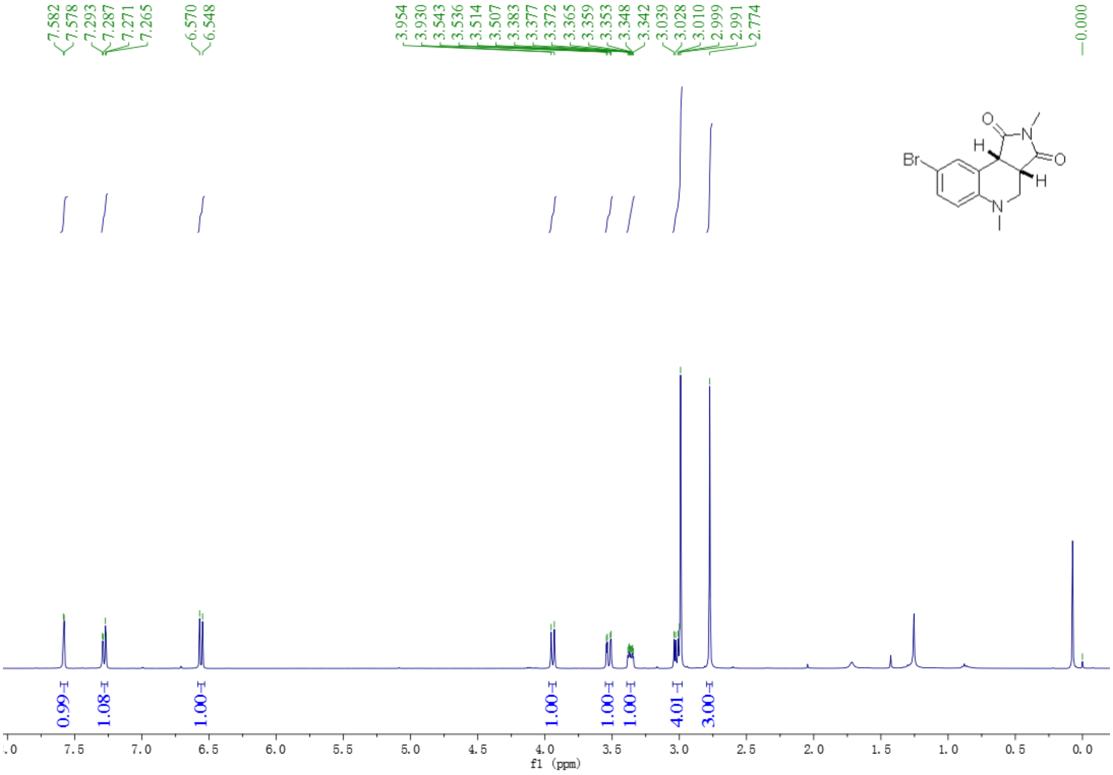
7.582
7.578
7.293
7.287
7.271
7.265
6.570
6.548
3.954
3.930
3.543
3.536
3.514
3.507
3.383
3.377
3.372
3.365
3.359
3.353
3.348
3.342
3.039
3.028
3.010
2.999
2.991
2.774
0.000
Br
H
O
N
O
H
N
0.99
1.08
1.00
1.00
1.00
1.00
4.01
3.00
f1 (ppm)

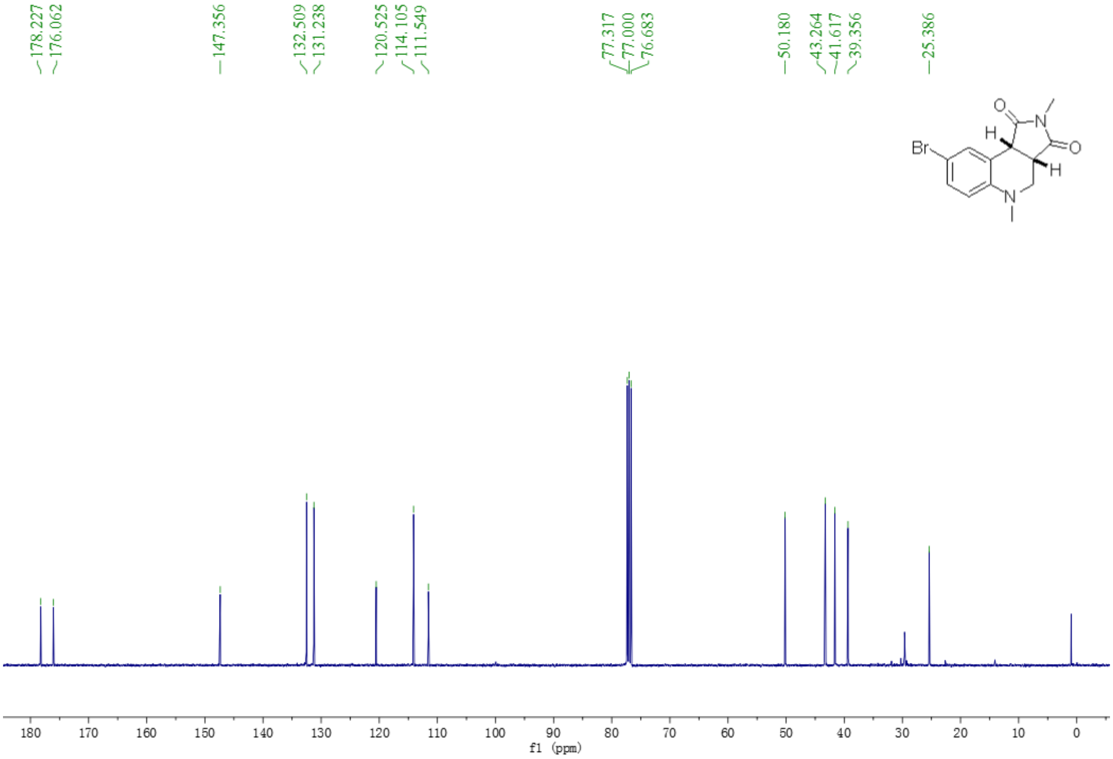
178.227
176.062
147.356
132.509
131.238
120.525
114.105
111.549
77.317
77.000
76.683
50.180
43.264
41.617
39.356
25.386
Br
H
O
N
O
H
N
f1 (ppm)

3p

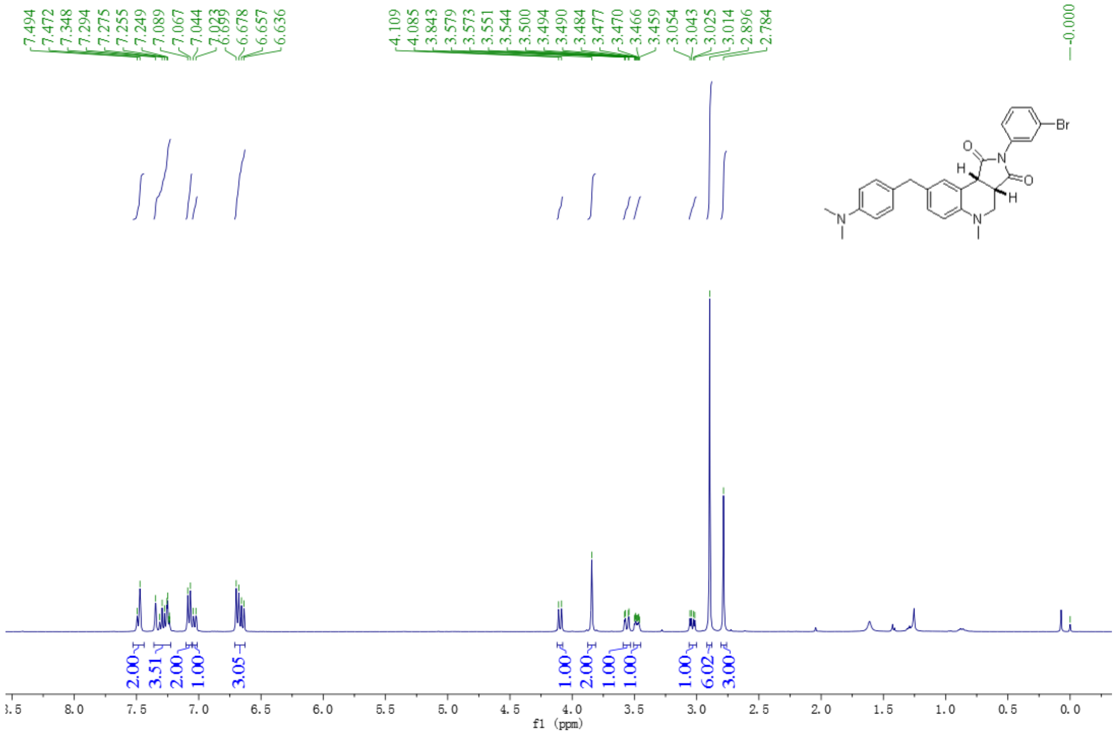
7.494
7.472
7.348
7.294
7.275
7.255
7.249
7.089
7.067
7.044
7.023
6.699
6.678
6.657
6.636
4.109
4.085
3.843
3.579
3.573
3.551
3.544
3.500
3.494
3.490
3.484
3.477
3.470
3.466
3.459
3.054
3.043
3.025
3.014
2.896
2.784
-0.000
Br
O
N
O
H
H
N
N
2.00
3.51
2.00
1.00
3.05
1.00
2.00
1.00
1.00
1.00
6.02
3.00
f1 (ppm)

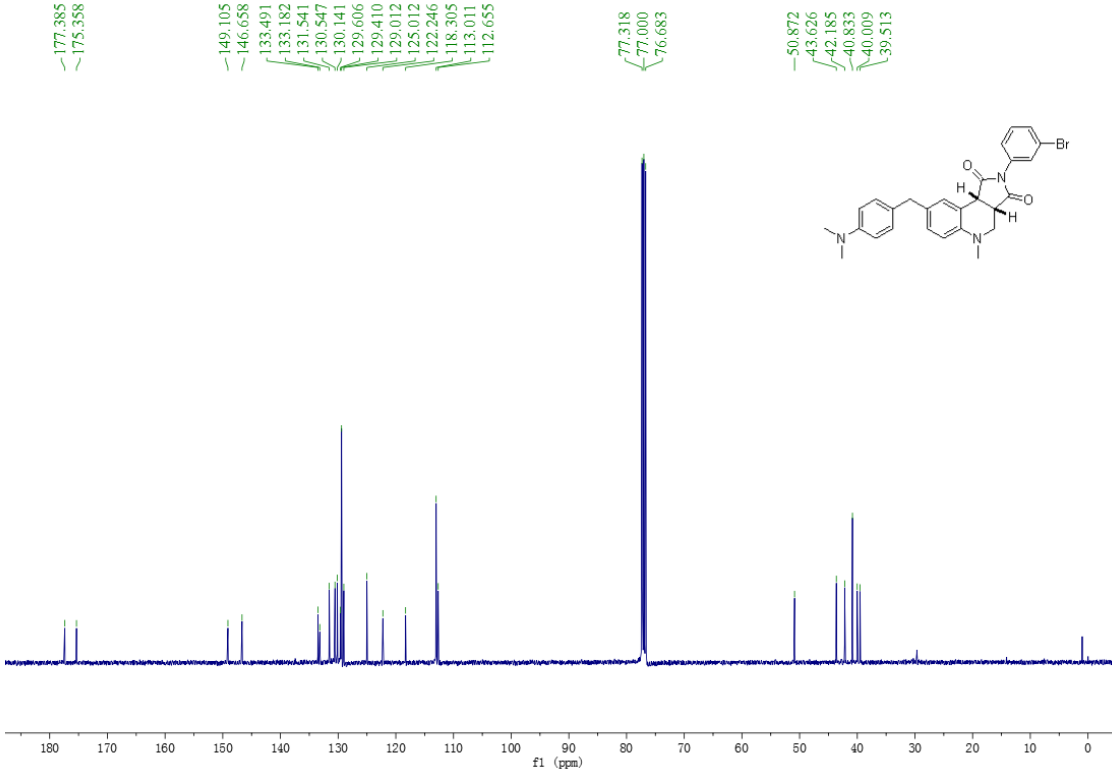
177.385
175.358
149.105
146.658
133.491
133.182
131.541
130.547
130.141
129.606
129.410
129.012
125.012
122.246
118.305
113.011
112.655
77.318
77.000
76.683
50.872
43.626
42.185
40.833
40.009
39.513
Br
O
N
O
H
H
N
N
f1 (ppm)

3q1+3q2

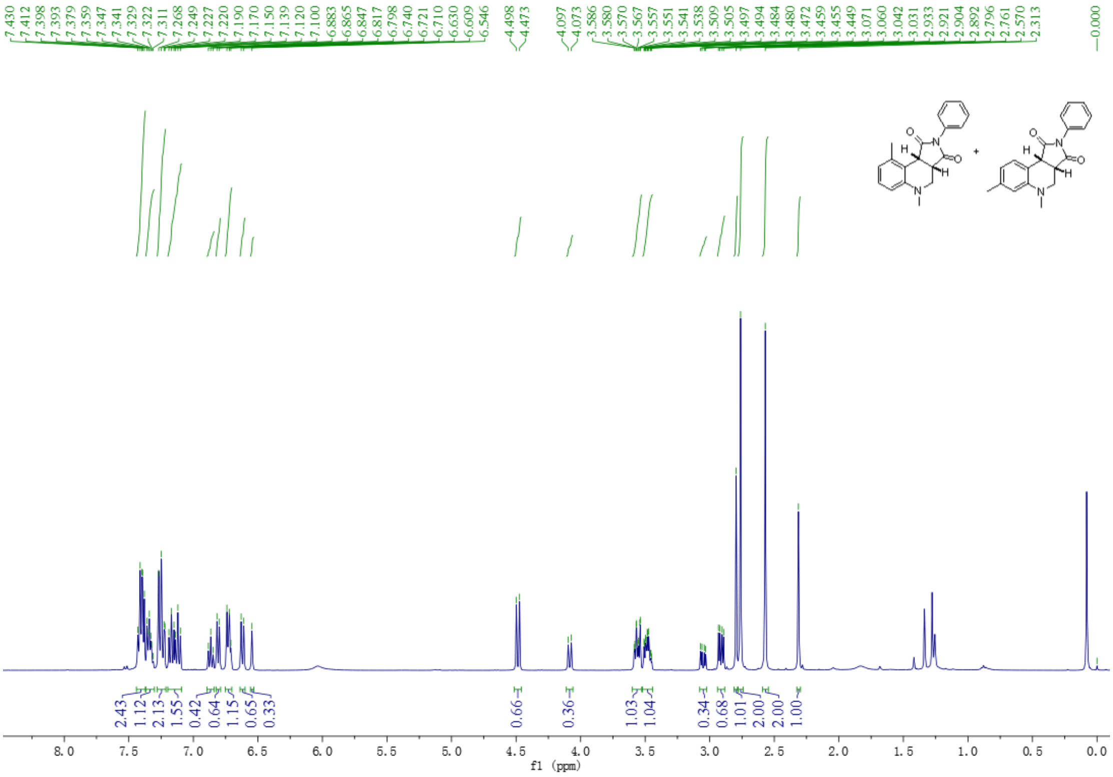

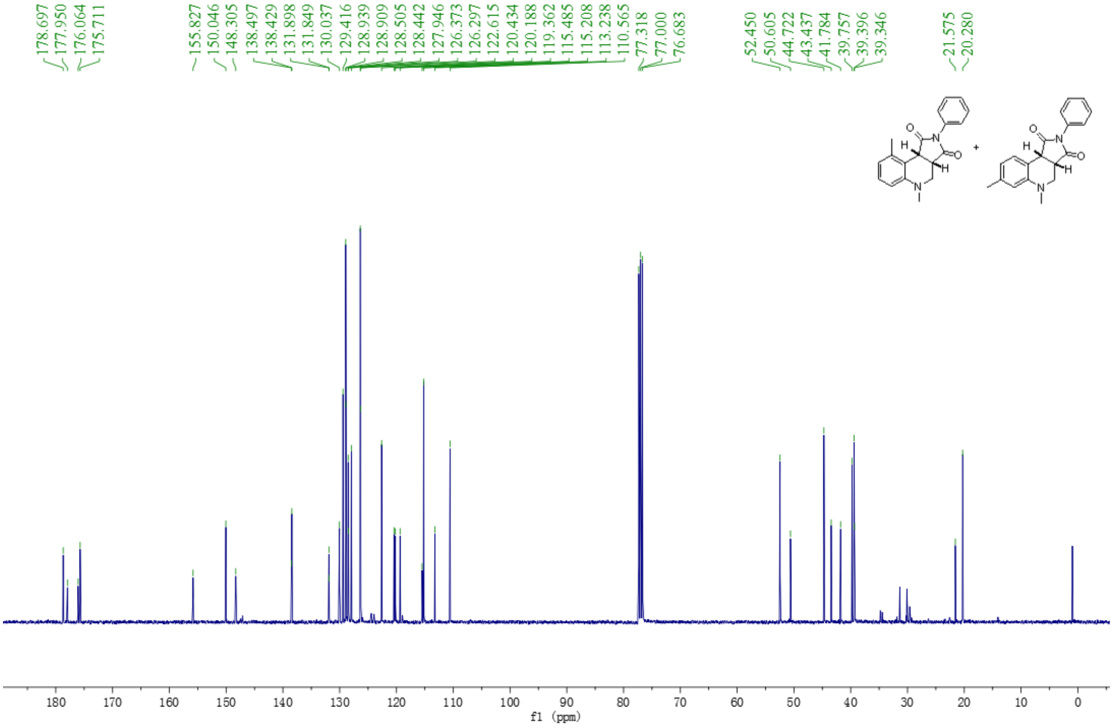

Supplement: File 1 — Experimental section and characterization of the synthesized compounds. [file Beilstein_J_Org_Chem-11-425-s001.pdf]
